# Major soluble proteome changes in *Deinococcus deserti* over the earliest stages following gamma-ray irradiation

**DOI:** 10.1186/1477-5956-11-3

**Published:** 2013-01-15

**Authors:** Alain Dedieu, Elodie Sahinovic, Philippe Guérin, Laurence Blanchard, Sylvain Fochesato, Bruno Meunier, Arjan de Groot, Jean Armengaud

**Affiliations:** 1 Laboratoire de Biochimie des Systèmes Perturbés, CEA Marcoule, DSV, iBEB, SBTN, LBSP, BAGNOLS-SUR-CEZE, F-30207, France; 2 CEA, DSV, IBEB, Lab Ecol Microb Rhizosphere & Environ Extrem (LEMiRE), Saint-Paul-lez-Durance, F-13108, France; 3 CNRS, UMR 6191, Biol Veget & Microbiol Environ, Saint-Paul-lez-Durance, F-13108, France; 4 Aix-Marseille Université, Saint-Paul-lez-Durance, F-13108, France; 5 INRA, UR1213 Herbivores, Saint Genès Champanelle, F-63122, France

**Keywords:** Proteome, Post-translational modification, Irradiation, Early response, Reference 2D map, Kinetics, Hierarchical clustering

## Abstract

**Background:**

*Deinococcus deserti* VCD115 has been isolated from Sahara surface sand. This radiotolerant bacterium represents an experimental model of choice to understand adaptation to harsh conditions encountered in hot arid deserts. We analysed the soluble proteome dynamics in this environmentally relevant model after exposure to 3 kGy gamma radiation, a non-lethal dose that generates massive DNA damages. For this, cells were harvested at different time lapses after irradiation and their soluble proteome contents have been analysed by 2-DE and mass spectrometry.

**Results:**

In the first stage of the time course we observed accumulation of DNA damage response protein DdrB (that shows the highest fold change ~11), SSB, and two different RecA proteins (RecA_P_ and RecA_C_). Induction of DNA repair protein PprA, DNA damage response protein DdrD and the two gyrase subunits (GyrA and GyrB) was also detected. A response regulator of the SarP family, a type II site-specific deoxyribonuclease and a putative N-acetyltransferase are three new proteins found to be induced. In a more delayed stage, we observed accumulation of several proteins related to central metabolism and protein turn-over, as well as helicase UvrD and novel forms of both gyrase subunits differing in terms of isoelectric point and molecular weight.

**Conclusions:**

Post-translational modifications of GyrA (N-terminal methionine removal and acetylation) have been evidenced and their significance discussed. We found that the Deide_02842 restriction enzyme, which is specifically found in *D. deserti*, is a new potential member of the radiation/desiccation response regulon, highlighting the specificities of *D. deserti* compared to the *D. radiodurans* model.

## Introduction

*Deinococcus deserti* belongs to the *Deinococcaceae*, a group of extremely radiotolerant bacteria. *D. deserti* VCD115 has been isolated from upper sand layers of the Sahara desert [1]. In this environment, cells are exposed to very changing life conditions with cycles of high temperatures, high UV irradiation, desiccating conditions, low temperatures and rehydrating conditions. Probably as a consequence of adaptation to such harsh, DNA-damaging conditions, *D. deserti* and other *Deinococcaceae*[2,3] exhibit an extraordinary ability to withstand ionizing radiation. Chromosomes with numerous radiation- or desiccation-induced double-strand breaks can be repaired in a few hours in *D. deserti*[4]. To learn about the specificities of this bacterium compared with other *Deinococcaceae*, its entire genome sequence has been determined [4]. It consists of a 2.8-Mb chromosome and three large plasmids called P1 (324 kb), P2 (314 kb), and P3 (396 kb). The comparative analysis showed some interesting differences between *D. deserti* and other sequenced *Deinococcus* species. For example, *D. deserti* possesses supplementary genes involved in DNA repair, such as three *recA* that code for two different RecA proteins, whereas it lacks homologs of several radiation-induced genes in *Deinococcus radiodurans* (e.g., *ddrP* encoding a putative DNA ligase).

The extreme radiotolerance of *Deinococcaceae* was the object of intense investigations during the last years using *D. radiodurans* as model. In cells subjected to irradiation, DNA recombinase RecA was the first protein that was found strongly induced [5]. RecA is essential for radiotolerance [6] and for the fidelity of DNA repair and genome stability in *D. radiodurans*[7]. The molecular mechanisms underlying DNA repair were also examined by transcriptomics [8-10] leading to the description of a repertoire of genes responding to acute gamma irradiation, including genes involved in DNA replication, repair and recombination, cell wall metabolism, cellular transport and many with uncharacterized functions. Another transcriptional study has shown that 72 genes were up-regulated three fold or higher in *D. radiodurans* following gamma irradiation [11]. In this study, besides genes with already known assigned function linked to DNA repair (*gyrA, gyrB, uvrA, uvrB, ruvB and recA*), five novel genes with unknown or hypothetical assigned functions were highlighted: *ddrA, ddrB, ddrC, ddrD and pprA* (ddr, DNA damage response; ppr, pleiotropic protein promoting DNA repair). Genetic analyses demonstrated a role of these five genes in radiotolerance [11], and further studies have reported that DdrA, DdrB and PprA are involved in DNA repair [12-19]. Radiotolerance of *D. radiodurans* probably results from a combination of different highly coordinated physiological pathways [20]. Besides enzymatic pathways such as DNA repair, passive contributions to radiotolerance have been described such as the nucleoid structure where DNA molecules are highly condensed, which would limit diffusion of DNA fragments [21], and the high intracellular Mn^2+^/Fe^2+^ ratio that limits protein oxidation [20,22-24]. Today, the survival kit components of *Deinococcaceae* are far to be exhaustive and some possible factors remain controversial [25].

To further analyze these mechanisms of radioresistance, proteomic approaches are promising as both mass spectrometry (MS) tools and protein separation techniques are ultimately quickly improving [26]. As gene expression and presence of the corresponding protein in the cell are not always strictly correlated, it is worth to compile multi-omics data on such subject [27]. Zhang *et al.*[28] identified by a classical 2D electrophoresis (2DE) gel approach 21 proteins whose cellular levels significantly changed following γ-irradiation of *D. radiodurans*. Surprisingly, among them few proteins are directly related to DNA metabolism. A comparative 2DE gel-based proteomic study, using the wild-type and a *pprI* knock-out strain, has shown that 31 proteins are up-regulated after exposure to low dose of γ radiation in a PprI-dependent manner in *D. radiodurans*[29]. PprI, also called IrrE, is a *Deinococcus*-specific transcriptional factor essential for radiotolerance [30-32]. Moreover, phosphorylation of two proteins (DR_A0283 which encodes a serine protease and DR_1343, a glyceraldehyde-3-phosphate dehydrogenase) was shown to be dependent upon the presence of PprI. More recently, a 2D-proteome reference map has been reported for *Deinococcus geothermalis* grown in standard conditions [33], as well as a shotgun analysis of its major membrane components [34].

Comparisons of the genome sequences of various *Deinococcaceae* shed new light on their potential arsenal related to DNA repair and radiotolerance [4,35,36]. Convinced that comparison of the proteome dynamics of several species will also be fruitful, we performed a time course analysis of the proteome of *D. deserti* after exposure to 3 kGy γ-radiations. The proteome from cells harvested at different time lapses after irradiation were analyzed with a 2-D gel approach. A hierarchical clustering analysis pointed at accumulated proteins at the earliest stages after irradiation. Among these are several important DNA repair proteins. Interestingly, post-translational modifications of GyrA were detected. We also observed the up-regulation of novel proteins such as the conserved Deide_20140 acetyltransferase, Deide_19260 response regulator, Deide_21840 PilT protein, as well as specific *D. deserti* proteins such as the Deide_02842 restriction enzyme. The physiological significance of these novel results is discussed.

## Results

### Time course analysis of *D. deserti* cells after drastic irradiation

**Figure 1:**
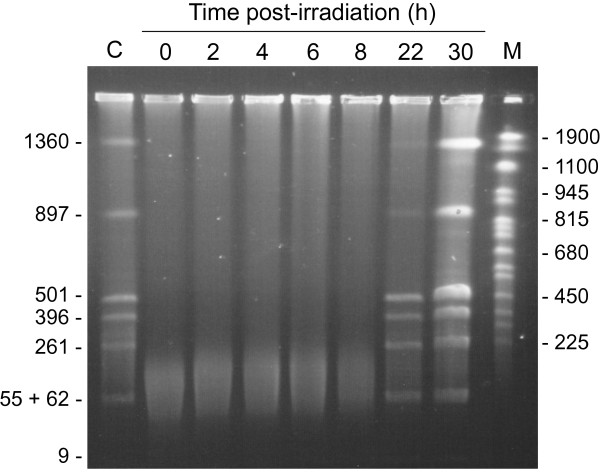
**Kinetics of genome reconstitution after gamma-irradiation by PFGE.** Genomic DNA was purified from cells before irradiation and at different times after irradiation, digested with PmeI and SwaI (resulting for an undamaged genome in 8 DNA fragments, of which the sizes (in kb) are indicated on the left, and separated by PFGE. Lane C, pre-irradiation control. Lane M, Yeast Chromosome PFG Marker (New England Biolabs). Lengths (in kb) of several marker fragments are indicated on the right.

We established a reference proteome map for *D. deserti* cells harvested at the exponential phase (Additional file 1: Figure S1 & Additional file 2: Table S1). We observed that several stress response and DNA repair proteins are already found in standard condition, for example two desiccation tolerance-associated proteins (Deide_07540, Deide_08510) and RecA_C_ (Deide_19450). We investigated the time-dependent changes in the proteome of *D. deserti* after drastic irradiation conditions. Two independent cultures of *D. deserti* VCD115 cells were grown overnight to reach an optical density of 0.4. Each culture was exposed to 3 kGy of gamma rays. After irradiation, each culture was incubated without change of growth medium to avoid any additional stress. Sampling took place immediately before irradiation and 0, 2, 4, 6, 8, 22 and 30 hours post-irradiation. We performed 2DE-gels from our two biological replicates with a specific effort in terms of technical replicates for time-points before irradiation and right after (0, 2 and 4 hours) in order to detect tiny changes in protein accumulation. In parallel to the 2DE-gel analysis, cell growth as well as DNA damage and repair was followed at the different time points. Figure 1 shows that while DNA is broken in small pieces by irradiation, DNA repair is slow in our experimental set-up with completion visible at 22 hours. Therefore, the early stages of DNA repair can be analyzed following the 2, 4, 6, and 8-hour time-points. No growth was observed for the first 22h (Additional file 3: Figure S2). The cells have started to grow only 30 h post-irradiation. This is in agreement with the PFGE data (Figure 1) that show an increased amount of DNA due to replication at 30 h.

### Proteins involved in response to drastic irradiation conditions

We determined the concentration of total proteins for each sample and deposited the same amount of proteins for each gel. We matched with the ImageMaster software the gels belonging to the same class, *i.e.* same time point. When comparing the different classes a significant trend was observed in terms of number of spots. The number of spots reported here is a mean value of the replicates. While before irradiation, roughly 488 (± 29) spots were delineated, only 467 (± 65) were detected at 0h, 458 (± 112) at 2h, 425 ((± 98) at 4h and 411 (± 3) at 6h and 410 (± 90) at 8 hours. An increase to 526(± 14) and 546 (± 100) spots was recorded for 22 and 30 hours, respectively. The minimum for both biological duplicates was reached 4–6 hours after irradiation. As it will be discussed below, the presence in large quantities of at least two proteases up-regulated in the first stages after irradiation could explain this phenomenon.

**Figure 2:**
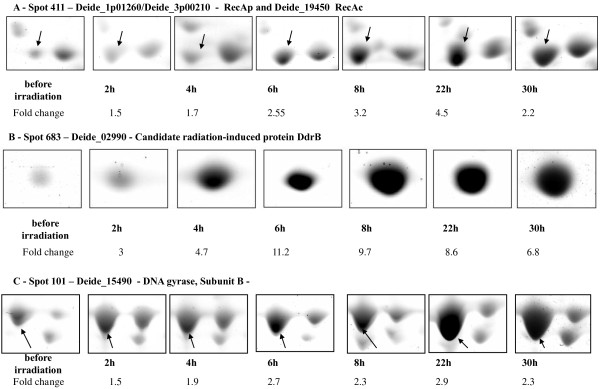
**Early enhanced production of proteins after drastic irradiation.** Protein spots in 2-D gels are shown for [**A**]: RecA_P_ (Deide_1p01260/Deide_3p00210) and RecA_C_ (Deide_19450), [**B**]: DdrB (Deide_02990), [**C**]: GyrB (Deide_15490). Images were taken from one gel, but the statistical significance of the fold change increase was calculated from a mean value of at least three gels.

After comparing gel images with the statistical tools implemented in the ImageMaster software, we detected five spots that were significantly increased in intensity after irradiation, with fold changes above 1.5 (p < 0.05). Figure 2 shows image captures of three of these spots and fold changes associated along the time courses. They are clearly more intense at 2, 4 and 6 hours and remain high at 8 and 22 h, which is in agreement with the kinetics of the genome recovery shown in Figure 1. We analyzed by MS the polypeptide content of each of these spots throughout the whole time course. The three series of spots shown in Figure 2 correspond to: Deide_1p01260/Deide_3p00210 (RecA_P_) and Deide_19450 (RecA_C_) in spot sp_411, Deide_02990 (DdrB; spot sp_683), and Deide_15490 (GyrB, subunit B of DNA gyrase; spot sp_101). The two others are Deide_00120 (SSB; spot sp_494) and Deide_2p01380 (PprA; spot sp_523) (data not shown). The two *recA* genes on plasmids P1 and P3 code for exactly the same protein named RecA_P_, which shares 81% sequence identity with the chromosome-encoded RecA_C_. They can be easily distinguished from each other with several proteotypic peptides (Additional file 4: Figure S3**)**. Under the spot sp_411, RecA_P_ is detected sooner along the time course and always in larger quantity compared with RecA_C_. These six proteins are all involved in DNA protection or repair as implied by several studies performed with the *D. radiodurans* model [8,11,37], or, for the different RecA proteins, with the *D. deserti* model [38]. Deide_02990 (DdrB) is clearly accumulating from 2 hours after irradiation (fold change ~ 3). Its presence is maximal at 6 hours (fold change ~11) in the time course whatever the biological replicate. The accumulation of Deide_00120 (SSB) is also detected early, as soon as 2 hours after irradiation. In this case, the fold change remains moderate (~ 2.5) till 8 hours. Abundance of RecA_P_, RecA_C_, GyrB, PprA is significantly increased only after 4 hours in our experimental set-up. We did not detect with confidence down-regulated proteins with such fold changes.

### Hierarchical clustering methodologies revealed novel key proteins in the early and delayed responses

**Figure 3:**
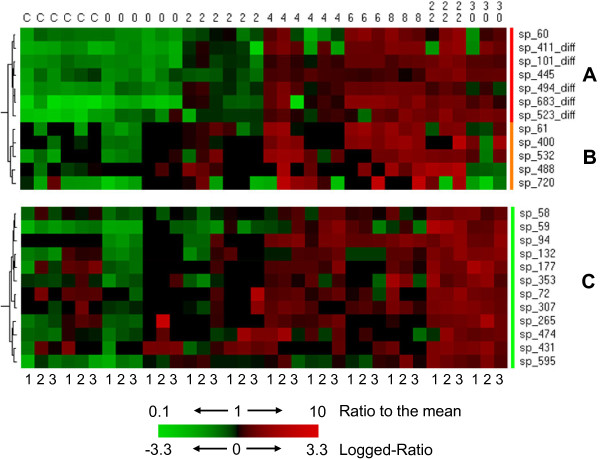
**Hierarchical clustering to identify early accumulating proteins.** Three clusters were selected from hierarchical clustering results. **A** (red cluster) and **B** (orange cluster) look very similar with up-regulated proteins at an early stage (2 hours after irradiation) but are statistically different. The red cluster contains 5 spots already detected by the statistic method implemented in the ImageMaster software (indicated with diff). The third one **C** (green cluster) gathers proteins up-regulated from 4 hours and beyond. The dendogram on the left indicates the order of the protein grouping. The intensities (protein accumulation) range from bright green (underproduced in comparison with the mean value) to bright red (overproduced in comparison with the mean value) according to the color scale at the bottom of the figure. The three successive columns for each biological sample correspond to the corresponding 3 technical replicates recorded and they are numbered in the bottom of the figure. Red and orange clusters are statistically different partly because of (i) missing values and (ii) inability of the statistical method available in the software program to analyze gel images (here, ImageMaster 2D) to detect these spots in some gels.

Clustering and heat map visualization was performed to identify groups of co-regulated proteins whose expression change over time. Figure 3 shows three clusters, namely red, orange and green clusters, corresponding to proteins accumulating at early stages: 2 h (high abundance increase), 2 h (less pronounced abundance increase), and 4 h after irradiation, respectively. We identified the polypeptides present in these spots and confirmed their presence along the whole time course if relevant. Table 1 presents the main characteristics of these proteins and the fold changes along the time course after irradiation. The “red” cluster comprises seven spots. Among these, five spots containing six proteins were already detected earlier in our direct gel *t*-test comparison. In the two additional spots, four new proteins were revealed: Deide_12520 (DNA gyrase subunit A) on the first hand, Deide_19260 (response regulator of the SarP family), Deide_20140 (GCN5-related N-acetyltransferase) and Deide_21840 (predicted twitching mobility protein) on the other.

**Table 1:** Radiation-induced proteins identified by hierarchical clustering analysis

**Spot**	**Protein identification**	**Protein description**	**Time (h) post irradiation**																	
			**2**			**4**			**6**			**8**			**22**			**30**		
			**FC (2/ct)**	**%VOL**	**t-test**	**FC (4/ct)**	**%VOL**	**t-test**	**FC (6/ct)**	**%VOL**	**t-test**	**FC (8/ct)**	**%VOL**	**t-test**	**FC (22/ct)**	**%VOL**	**t-test**	**FC (30/ct)**	**%VOL**	**t-test**
sp_60	Deide_12520	DNA gyrase, subunit A	1,64	0,14	<0,01	1,84	0,16	0,02	3,89	0,34	<0,01	3,59	0,31	<0,01	3,68	0,32	<0,01	2,00	0,18	<0,01
sp_101_diff	Deide_15490	DNA gyrase, subunit B	1,82	0,39	<0,01	2,44	0,53	<0,01	3,36	0,72	<0,01	3,07	0,66	<0,01	3,93	0,85	<0,01	2,85	0,62	<0,01
sp_411_diff	Deide_1p01260/ Deide_3p00210	RecA_P_	2,28	0,16	<0,01	3,31	0,23	<0,01	4,51	0,31	<0,01	5,62	0,39	<0,01	4,91	0,34	<0,01	3,74	0,26	<0,01
sp_445	Deide_20140	GCN5-related N-acetyltransferase	1,29	0,08	0,09	2,21	0,14	<0,01	2,06	0,13	<0,01	2,03	0,13	<0,01	3,35	0,21	<0,01	2,67	0,17	<0,01
sp_445	Deide_19260	response regulator, SarP	1,29	0,08	0,09	2,21	0,14	<0,01	2,06	0,13	<0,01	2,03	0,13	<0,01	3,35	0,21	<0,01	2,67	0,17	<0,01
sp_445	Deide_21840	twitching mobility protein	1,29	0,08	0,09	2,21	0,14	<0,01	2,06	0,13	<0,01	2,03	0,13	<0,01	3,35	0,21	<0,01	2,67	0,17	<0,01
sp_494_diff	Deide_00120	single-stranded DNA-binding protein	2,05	0,37	<0,01	2,85	0,52	<0,01	3,40	0,62	<0,01	3,63	0,66	<0,01	2,84	0,52	<0,01	2,07	0,38	<0,01
sp_523_diff	Deide_2p01380	DNA repair protein PprA	1,81	0,14	0,05	2,66	0,21	<0,01	2,50	0,20	0,04	4,05	0,32	<0,01	4,32	0,34	<0,01	4,13	0,33	<0,01
sp_683_diff	Deide_02990	DNA damage response protein DdrB	4,50	0,57	<0,01	5,99	0,76	<0,01	11,98	1,52	<0,01	10,51	1,33	<0,01	8,86	1,12	<0,01	6,83	0,87	<0,01
sp_61	Deide_12520	DNA gyrase, subunit A	2,51	0,07	<0,01	4,77	0,13	0,01	5,49	0,15	<0,01	5,10	0,14	<0,01	3,66	0,10	0,01	2,42	0,07	0,01
sp_400	Deide_19450	RecA_C_	1,55	0,28	0,06	3,05	0,55	<0,01	3,12	0,57	<0,01	3,69	0,67	<0,01	5,92	1,07	<0,01	2,64	0,48	ns
sp_488	Deide_13740	signal recognition particle-docking protein FtsY	1,69	0,03	0,12	2,66	0,05	0,06	2,45	0,04	0,01	2,58	0,04	0,02	3,89	0,07	<0,01	0,75	0,01	ns
sp_532	Deide_02842	Type II site-specific deoxyribonuclease	2,13	0,06	0,06	3,22	0,09	0,01	3,10	0,09	<0,01	3,54	0,10	<0,01	3,26	0,09	<0,01	2,04	0,06	0,06
sp_532	Deide_14090	LAO/AO transport system kinase	2,13	0,06	0,06	3,22	0,09	0,01	3,10	0,09	<0,01	3,54	0,10	<0,01	3,26	0,09	<0,01	2,04	0,06	0,06
sp_720	Deide_01160	DNA damage response protein DdrD	1,48	0,07	ns	5,00	0,23	0,03	10,23	0,46	<0,01	10,24	0,46	<0,01	3,29	0,15	0,19	0,67	0,03	ns
sp_58	Deide_19590	ATP-dependent protease La	1,06	0,07	ns	1,43	0,09	0,01	1,28	0,09	0,14	1,34	0,09	0,08	2,06	0,14	<0,01	1,85	0,12	<0,01
sp_58	Deide_12520	DNA gyrase, subunit A	1,06	0,07	ns	1,43	0,09	0,01	1,28	0,09	0,14	1,34	0,09	0,08	2,06	0,14	<0,01	1,85	0,12	<0,01
sp_59	Deide_12520	DNA gyrase, subunit A	1,45	0,06	ns	2,96	0,12	<0,01	2,28	0,09	0,01	4,60	0,18	0,04	6,60	0,26	<0,01	3,67	0,14	<0,01
sp_72	Deide_12100	DNA helicase II (UvrD)	1,16	0,03	ns	1,20	0,03	0,25	0,77	0,02	ns	7,62	0,18	0,10	1,56	0,04	0,04	1,23	0,03	ns
sp_94	Deide_15490	DNA gyrase, subunit B	1,95	0,04	ns	3,80	0,07	<0,01	3,28	0,06	0,01	4,11	0,08	<0,01	4,45	0,09	0,01	4,07	0,08	0,03
sp_132	Deide_02310	dipeptidyl aminopeptidase	1,19	0,04	ns	1,63	0,06	0,01	1,12	0,04	ns	1,73	0,06	0,03	2,62	0,09	<0,01	2,61	0,09	<0,01
sp_307	Deide_23290	GTP-binding protein	1,30	0,07	ns	1,34	0,07	ns	1,12	0,06	ns	1,64	0,09	0,10	1,83	0,10	0,05	1,64	0,09	0,12
sp_353	Deide_16440	biotin synthase/thiamine biosynthesis enzyme	0,79	0,02	ns	1,29	0,04	0,07	5,14	0,15	0,03	2,21	0,07	0,06	2,12	0,06	<0,01	2,17	0,06	<0,01
sp_474	Deide_1p00780	flavin dependant oxidoreductase	1,78	0,04	0,10	1,70	0,04	0,06	1,83	0,04	0,02	1,63	0,04	0,07	1,88	0,04	0,05	1,78	0,04	0,01
sp_595	Deide_15960	NADH dehydrogenase	1,72	0,06	0,02	1,60	0,06	0,04	1,55	0,05	ns	2,18	0,08	0,01	2,82	0,10	<0,01	2,58	0,09	<0,01

FC, fold change; %VOL, averaged spot intensity calculated as the relative volume; ns, not significant; ct, control (average of intensity at time 0 and before irradiation).

When several proteins were identified in the same gel spot, the number of peptides, the percentage of sequence coverage, and the emPAI score of each protein were analysed. If the variation of these parameters were identical between the identified abundant proteins, the global fold change of the spot was assigned to these proteins.

The “orange” cluster comprises five spots slightly more intense from 2 hours after irradiation, but not as much as the former cluster. In these five spots we could identify six up-regulated proteins (Table 1): Deide_12520 (DNA gyrase subunit A) which appeared previously in the “red” cluster in a clearly distinguishable spot, Deide_19450 (chromosome-encoded RecA_C_), Deide_02842 (a protein related to Type II site-specific deoxyribonuclease), Deide_14090 (a kinase involved in LAO/AO transport system), Deide_13740 (signal recognition particle-docking protein FtsY/cell division protein FtsY), and Deide_01160 (homologue of the *D. radiodurans* radiation-induced protein DdrD). In spot sp_400 (orange cluster), RecA_C_ is detected by many proteotypic peptides.

The “green” cluster comprises nine spots whose intensities slightly increase from 4 hours. From these spots we could identify 9 different up-regulated proteins (Table 1). Among these, several proteins are related to central metabolism (Deide_16440, Deide_1p00780, Deide_15960) and protein turn-over (the Deide_19590 La protease and Deide_02310 dipeptidyl aminopeptidase). Remarkably, we found within this cluster three novel spots corresponding to the gyrase subunits, Deide_12520 and Deide_15490. We also found up-regulation of DNA helicase UvrD (Deide_12100). UvrD plays a major role in Double-Strand Break repair through Extended Synthesis-Dependent Strand Annealing [39]. Last but not least, we found the EngA GTPase (Deide_23290) that was shown essential for cell viability in *E. coli* and required for ribosome assembly and stability [40].

### Evidences for post-translational modifications of GyrA N-terminus

**Figure 4:**
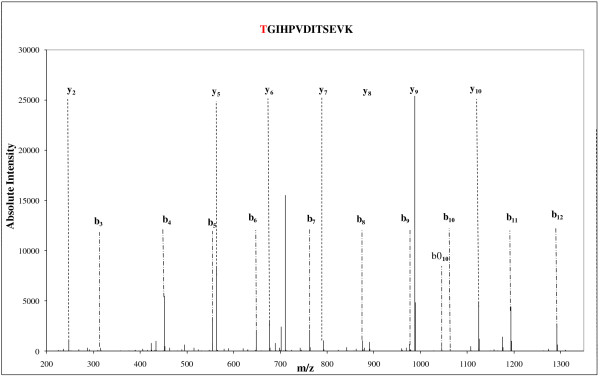
**MS/MS evidences for post-translational modifications of the most N-terminal peptide of GyrA.** The figure shows the MS/MS spectrum of a 2^+^ charged peptide ion at 719.38 *m/z* corresponding to the sequence [TGIHPVDITSEVK] with an acetylation at its N-terminus. As shown in the spectrum, all the main peaks correspond to the complementary *y* and *b* ion series of this modified GyrA most-N terminal peptide.

We detected several distinguishable spots (spots sp_58, sp_59, sp_60, and sp_61) belonging to the three different HCA clusters for the subunit A of gyrase (Deide_12520). Whether a post-translational modification such as Ser-Thr-Tyr phosphorylation, Lys or N-terminus acetylation, methylation, or maturation by proteases could explain this behaviour was investigated. In these spots, we found that the GyrA polypeptide is modified with the removal of its first residue (methionine) as can be expected because the second residue in the unmatured polypeptidic chain has a small lateral chain. Figure 4 shows the MS/MS spectrum of the N-terminal most peptide [TGIHPVDITSEVK] resulting from this specific modification. Moreover, for the spectrum of this peptide obtained with a LTQ-Orbitrap-XL mass spectrometer, the *b*_*3*_ ion with a *m/z* value of 314.17 indicated that an acetylation occurs on the TGI N-terminal tripeptide while the parent mass is unambiguously determined with a precision below 1 ppm. We noted that the first threonine residue can be partially acetylated as another MS/MS spectrum corresponded to the unacetylated [TGIHPVDITSEVK] form, with a *m/z* of the parent at 632.33 and a charge of 2^+^. A pI shift for acidic or neutral proteins due to acetylation has been observed [41,42]. While a mixture of acetylated peptide and non-modified peptide is seen in spots sp_59, sp_60, and sp_61, only the acetylated form is detected in spot sp_58. While this post-translational modification could explain the shift for spot sp_58, other differences between the three other spots should come from additional post-translational modifications. Interestingly, the up-regulation of Deide_20140 (a putative N-acetyl transferase) as presented in Table 1 could explain differences in acetylation pattern.

## Discussion

Following the proteome dynamics of *D. deserti* after irradiation by a 2DE-gel approach highlighted the up-regulation of 21 proteins. Among these, ten proteins either have no homolog in *D. radiodurans* or were not found to be radiation-induced at the protein or RNA level in this organism (see Additional file 5: Table S2 and below), whereas eight others are known to be important players of the irradiation response in *D. radiodurans*: DdrB, SSB, PprA, RecA, GyrA/B, UvrD and DdrD. Concerning RecA, a notable difference is that two functionally different RecA proteins are up-regulated in *D. deserti*. The other sequenced *Deinococcus* species possess only one *recA* gene. Both RecA_C_ and RecA_P_ contribute to radiotolerance, but only RecA_C_ is able to induce expression of DNA translesion polymerases in *D. deserti*[38]. The proteomic results obtained by Zhang *et al.*[28] from the 2D-gel based analysis of *D. radiodurans* after irradiation did not reveal the presence of GyrB and RecA while transcriptomics revealed the up-regulation of their genes [11] (Additional file 5: Table S2). Lu *et al.*[29] did see RecA induction, but apparently not GyrA, GyrB, DdrB, and DdrD accumulation. Here, we found a good correlation with the transcriptomics data obtained with *D. radiodurans* because GyrA, GyrB, RecA, DdrB and DdrD polypeptides are found accumulating in large quantities from 2 hours after irradiation. While this work was in progress, a new proteomic study with *D. radiodurans* was published, also showing up-regulation of GyrA, GyrB and DdrB, but not of DdrD (Additional file 5: Table S2). Using a 2DE-gel approach, Basu & Apte [37] identified ten and two different forms of induced SSB and DdrB, respectively, with each form in a different spot. These different forms are likely resulting from proteolytic processing of the C-terminal end of these proteins [37]. In our study with *D. deserti*, we identified up-regulated SSB and DdrB in only one spot each. Interestingly, we found different spots for both GyrA and GyrB (see below). The use of different bacterial species and experimental conditions are likely explanations for the differences between our results and those previously published on *D. radiodurans*[37].

Remarkably, we found the presence of several spots assigned to the same protein: four (spots sp_58, sp_59, sp_60, sp_61) and two (spots sp_101, sp_94) spots correspond to the GyrA and GyrB subunits, respectively. The different forms of Deide_12520 (GyrA) detected in the three HCA clusters are produced in higher amount after irradiation. The GyrA spots (spots sp_58, sp_59, sp_60, and sp_61) are only separated by small p*I* shifts. As reported previously by Zhu et al. [42], shifts in p*I* often correlate to protein modifications. These modifications include truncations or deletions, but also post-translational modifications of amino acid lateral chains such as phosphorylation or acetylation. They can also be due in a limited number of cases to alternative translation starts as revealed in an extensive survey of N-termini of proteins from *D. deserti*[43], or N-terminal trimming by exo-peptidases. At the N-terminus and side chains of amino groups, post-translational modifications will lead generally to small (<0.2) isoelectric point shift. Here, we observed a sequence coverage of ~30% with the GyrA peptides in the four spots. We detected the [2-TGIHPVDITSEV-13] and the [790-VINIAERDSVISAFPIRR-807] peptides that are very close from the expected N-terminal and C-terminal extremities of the 811 amino acid-long GyrA. For this reason, a small truncation of the protein is unlikely, except maturation of the N-terminus (removal of the initial methionine) as evidenced by the most-N-terminal peptide. Regarding post-translational modification of GyrA, we could not detect any phosphorylation for serine, threonine or tyrosine residues, nor acetylation of the lysine side chains within the detected peptides. However, we found that GyrA N-terminus is acetylated. While a mixture of acetylated peptide and non-modified peptide is seen in spots sp_59, sp_60, and sp_61, only the acetylated form is detected in spot sp_58. For eukaryotic proteins acetylation is one of the most common covalent modifications with phosphorylation [44] while it is not frequent for prokaryotic proteins [45,46] even if prokaryotic proteins are also extensively post-translationally modified [47]. Amino-terminal acetylation occurs co-translationally with eukaryotic proteins [45] and post-translationally in the case of prokaryotic proteins [48]. Acetylation may affect protein functions as their stability and DNA binding activity may be modified; the hyperacetylation of histones being the most illustrative example in this respect as it influences drastically transcription [49]. Hwang *et al.*[50] noted that N-terminal acetylation of proteins could act as a degradation signal in yeast. Interestingly, we found the Deide_20140 GCN5-related acetyltransferase being up-regulated in the earliest time-points after irradiation. Whether this enzyme is responsible for the acetylation observed on GyrA is an interesting hypothesis that deserves further investigation. However, at least under our experimental conditions, a potential Deide_20140-specific function is not essential for radiation resistance, since a *Deide_20140* deletion mutant appeared to be as radiotolerant as the wild-type strain (data not shown). The *D. deserti* genome encodes various putative N-acetyltransferases, and some of these might have redundant activities.

We observed two up-regulated proteases: Deide_19590 (protease La, also called Lon protease) and Deide_02310 (related to dipeptidyl-aminopeptidase/acylaminoacyl-peptidase). Of these, Deide_02310 is not conserved in other sequenced *Deinococcus* species. The presence of these proteases in significant amounts is delayed compared to most DNA-repair related proteins. Their up-regulation, together with the presence of several other proteases (Additional file 2: Table S1) [4], could explain the significant trend observed in terms of number of spots detected in our 2DE-gels. A very active protein turn-over occurs because of protein damages and intense metabolic changes. Protein degradation after exposure to radiation was also reported for *D. radiodurans*[51], which also encodes a high number of proteases. Other data indicated that Lon proteases play a key role in degradation of damaged proteins in *D. radiodurans*[52]. Proteolysis may also control the levels of radiation-induced proteins, as shown for *E. coli* where numerous SOS response proteins, including RecA and UvrA, are substrates for ClpPX, Lon and other proteases [53,54]. Accumulated levels of some DNA repair proteins can be deleterious, and their activity must be restricted to regions of DNA damage. Translesion DNA polymerases are other examples of induced proteins that are rapidly degraded *in vivo*[54]. *D. deserti* encodes functional translesion DNA polymerases that are induced upon DNA damage [38], and their activity should be strictly controlled to prevent elevated levels of mutagenesis. Remarkably, the regulation of the dipeptidyl-aminopeptidase/acylaminoacyl-peptidase (Deide_02310) could explain the heterogeneity observed for the GyrA/B subunits.

Besides the already known proteins related to DNA-repair or metabolic changes described above, we detected the presence of several novel proteins that probably fulfill key roles in the radiation responses in *D. deserti.* Deide_20140 presents some far-related similarities with MshD (E value: 3.01e-09), the acetyl-transferase that catalyzes the final step of mycothiol biosynthesis in various members of the Actinomycetes. Mycothiol replaces glutathione in these species. Glutathione is a well-known antioxidant that helps protecting cells from reactive oxygen species such as free radicals and peroxides. Together with other antioxidants, such as Mn^2+^ complexes [5,22], the Deide_20140 acetyl-transferase in *D. deserti* could contribute to tolerance to oxidative stress, which is generated by ionizing radiation-induced water radiolysis [55]. Oxidative stress may also occur during dehydratation as described by Fredrickson and co-workers [56]. Interestingly, we observed the up-regulation at the earliest stage after irradiation of the Deide_19260 protein (COG3947) that shows strong similarities with response regulators from two-component systems. COG3947 members usually comprise two structural domains: i) at their N-terminus, a CheY-like receiver with a phosphoacceptor site (Asp52 in Deide_19260) that is phosphorylated by histidine kinase homologs and ii) a DNA-binding transcriptional activator of the SARP family at their C-terminus. We tried to check the phosphorylation status of the CheY domain, but a peptide covering residue 52 was not detected in our experiments. Such aspartate-phosphorylation should be stable enough to be detected in our experimental conditions as previously found [57,58].

It is worth to note that this putative two-component regulator is highly conserved in all radiotolerant *Deinococcus* species (70–84% identity) and in the thermophilic and radiotolerant *Truepera radiovictrix* (41% identity). However, the genetic context for *Deide_19260* and its homologs is not conserved. None of the genes flanking *Deide_19260* or its homologs encodes a histidine kinase in these species. The putative cognate histidine kinase for Deide_19260 is unknown. The Deide_19260 protein could be an important regulator for stress and/or DNA-damage response in *D. deserti*, besides the previously identified IrrE transcriptional activator. It will be of interest to study the targets of the SARP regulator. However, in contrast to the radiation-sensitive *irrE* mutant [32], deletion of *Deide_19260* did not result in loss of radiation tolerance (data not shown). This could mean that Deide_19260 is not involved in radiation tolerance or that its function is redundant with another response regulator. The Deide_21840 up-regulated protein is related to PilT (COG2805), a nucleotide binding protein responsible for the retraction of type IV pili, likely by pili disassembly. This retraction provides the force required for travel of bacteria in low water environments. This protein is also required for DNA uptake in several bacteria [59]. Three proposed roles for DNA uptake are genetic transformation, DNA repair, and to provide a source of nutrient [60]. PilT may thus be an important element for survival at the population level of *D. deserti* upon adverse conditions. Another up-regulated protein, Deide_02842, presents far-related similarities with BglI restriction enzyme of *Bacillus atrophaeus*, previously known as *Bacillus globigii*[61,62]. We can predict that, like BglI, Deide_02842 is also a type-II site-specific ribonuclease, *i.e.* it cleaves specifically within or close to the recognition sequence in DNA. The *Deide_02841* gene predicted to encode a DNA methylase is adjacent to *Deide_02842*. DNA methylation by this enzyme would protect *D. deserti*’s own DNA from cleavage by Deide_02842. Homologs of both genes are absent from other sequenced *Deinococcus* species. *D. deserti* may have acquired these genes by horizontal gene transfer, as suggested by their low GC percentage (46 and 50% versus an average of 63% for the total genome) and the nearby located transposase and integrase genes. Such restriction enzyme and methylase duo is known to be involved in the protection of bacterial cells by limiting incorporation of incoming foreign DNA, such as from bacteriophages, into the host genome. The Deide_13740 up-regulated protein (FtsY signal recognition particle GTPase) constitutes a universally conserved protein targeting pathway that ensures the co-translational delivery of substrates to the membrane-bound Sec translocon [63]. Logically, the delivery of proteins synthesized in the cytosol to their correct cellular compartment is of utmost importance for the cell following gamma-irradiation. Deide_14090 shows strong sequence similarities with the ArgK kinase that phosphorylates periplasmic binding proteins involved in the LAO (lysine, arginine, ornithine)/AO transport systems [64].

To investigate if the proteins identified in this study may be up-regulated by a common mechanism, the upstream regions of the corresponding 22 genes were analyzed using MEME [65]. A 17-bp palindromic motif was found upstream of 11 genes: *gyrA*, *gyrB*, *ssb*, *pprA*, *ddrB*, *ddrD*, *uvrD*, *recA*_C_, *recA*_P1_, *recA*_P3_ and *Deide_02842* (Type II restriction enzyme). This motif corresponds to the radiation/desiccation response motif (RDRM) first identified after analysis of radiation-induced genes in *D. radiodurans* and *D. geothermalis*[36]*.* In a previous study we scanned the entire genome of *D. deserti* with this motif and a match with the RDRM was found in the upstream region of 25 genes, including 10 of the 11 genes mentioned above [4]. Here, using another method (MEME), *Deide_02842* is found as a new potential member of the RDRM regulon [4,36]. It has been shown that radiation-induced transcription of at least some of these RDRM-containing genes is dependent on PprI (IrrE) [30,31,38]. Besides the RDRM upstream of 11 genes, no other motifs were found for the up-regulated proteins identified here, and transcription of the other 11 genes may be regulated in different manners. Alternatively, accumulation of the corresponding proteins may not be related to up-regulated transcription but rather to an increase of their translation and/or to protein-protein or protein-DNA interactions that increase their stability.

We have shown that DdrB and SSB are clearly accumulating in large quantities in the very earliest stages after irradiation, at least in our experimental conditions. These two single-stranded DNA binding proteins are probably of high importance to protect ssDNA that is formed after massive DNA damage and/or to initiate genome reconstitution by recruitment of other DNA repair proteins. That they are both up-regulated at the same early stage may indicate that they work concomitantly. Interestingly, Xu et al. [19] have recently shown that both proteins interact *in vitro*. Another recent study reported that DdrB is involved in DNA repair through a single-strand annealing (SSA) process that precedes the Extended Synthesis-Dependent Strand Annealing (ESDSA) [12]. Finally, the specific post-translational modifications of GyrA detected from irradiated samples raises the importance of post-translational modifications of the *Deinococcus* proteome upon DNA damages. Whether the resulting heterogeneity impacts the cellular response is an open question.

## Materials and methods

### *D. deserti* cellular growth and irradiation conditions

*D. deserti* strain VCD115 [1] was grown in 10-fold diluted tryptic soy broth (TSB/10) supplemented with trace elements [32]. To prevent other fluctuations than the irradiation, pre- and post-irradiation incubation was performed under conditions similar to the irradiation ones, *i.e.* 20°C and without vigourous shaking. Two independent 1L cultures were grown to exponential phase (OD_600_ 0.4) and exposed to 3 kGy of gamma rays (39 Gy/min, ^60^Co source; CEA Cadarache, France). This resulted in 68% survival as determined by plating of serial dilutions and colony-forming unit counting. Samples of 100–150 ml for proteome analysis were taken immediately before irradiation and at different time points after irradiation. Cells were harvested by centrifugation, washed twice with 50 mM Tris–HCl pH 8.0, frozen in liquid nitrogen and stored at −80°C. Culture samples (5 ml) were also taken for pulsed-field gel electrophoresis (PFGE) analysis. PFGE analysis of genome reconstitution was performed as described [4].

### *D. deserti* protein extracts

For each condition, cells (0.2 g of wet material) were resuspended in 3 mL of lysis buffer consisting of 8.75 M urea, 2.5 M thiourea, 50 mM DTT, 25 mM spermine, and 5% CHAPS, and containing a protease inhibitor cocktail (Complete Mini) from Roche (1 tablet per sample). Cells were disrupted with an ultrasound Vibracell 75042 sonicator (Fisher Bioblock Scientific) for 35 sec (7 cycles of 5 sec pulse and 5 sec pause). The extracts were then ultracentrifuged for 1h at 100,000 *g* at 4°C to remove cellular debris. Protein concentration from the supernatants was determined by a Bradford assay (Biorad) using Bovine Serum Albumin as a standard after diluting the samples to perform the assay below the limit of 4M urea.

### 2-D electrophoresis and image analysis

Because we followed several points along the time course after irradiation, only two independent biological replicates were performed. Because numerous biological replicates are difficult to perform regarding the radiation facilities used, specific effort in term of technical replicates has been done especially for time-points before irradiation and right after (0, 2 and 4 hours) with 4 gels done per biological replicate for these time-points while 2 gels were done per biological replicate at 6, 8, 22 and 30 hours, resulting in a total of 48 gels for the whole analysis. Immobilines 18 cm Drystrips pH 3–10 (GE Healthcare) were rehydrated overnight with 300 μg of protein extract. Isoelectric focusing was performed with a Multiphor system (GE Healthcare) up to 60 kVh. After focusing, separation strips were equilibrated for 15 min in 50 mM Tris–HCl buffered at pH 8.8 and containing 6 M urea, 30% glycerol, and 10 mg/ml dithiothreitol to disrupt disulphide bridges. The reduced thiols were then alkylated with 25 mg/ml iodoacetamide in the same buffer for 15 min. The second dimension was performed using 12 % acrylamide gels in Protean II xi 2-D cell (Biorad) at 25V for 1h then at 12.5 W/gel. The gels were first fixed in 5% acetic acid and 30% ethanol overnight, washed 3 times in H_2_O for 10 min, treated with 0.02% sodium thiosulphate for 1 min, and then rinsed twice with H_2_O for 1 min. The gels were stained overnight with Coomassie Brilliant Blue G250 (Biorad) and then rinsed twice with distilled water. These gels were scanned using an Umax Scanner (Pharmacia) at 600 dpi. Image analysis was performed using ImageMaster 2D Platinum v5.0 (GE Healthcare). The protein content of each spot was determined by its relative volume compared with the sum of all spot volumes in the gel, and expressed as a percentage of volume (%Vol). The replicated gels corresponding to the same time point were grouped in the same class. Statistical analysis was performed on individual sample classes, the control class comprising gels where unirradiated samples were resolved. Class ratios above 1.5 (meaning 1.5 fold change compared with control) were selected, t-test controlled (p<0.05), and the differences were examined visually prior to identification by MS. For the 2DE reference map, protein spots were identified by peptide mass fingerprint using a Biflex IV mass spectrometer (Bruker Daltonics). Some faint spots were identified by tandem mass spectrometry done with an Esquire ion trap (Bruker Daltonics) coupled to an Ultimate™ NanoLC system (Dionex-LC Packings) . For the time-course experiment, protein spots were identified by tandem mass spectrometry done with an LTQ-Orbitrap XL (Thermo) mass spectrometer.

### Hierarchical clustering analysis (HCA)

HCA was used to group proteins with similar expression profile and help for visual selection of interesting clusters as described [66]. To this end, the complete %Vol matrix from image analysis was logged-ratio transformed (i.e. each protein profile centered on its mean value after being logged) and imported in the PermutMatrix software [67]. HCA was then processed according to the Pearson distance and the Ward aggregation procedure to construct the resulting dendrogram and to allow heat map visualization of the clustered data matrix [66]. Only clusters with interesting profile regarding reconstitution of intact DNA molecules, i.e. those with spots intensity clearly increasing during the early time-course experiment (time 2, 4, 6 and 8 h post irradiation), were selected. The corresponding spots were manually controlled for missing values and/or bad alignments so that false positive detection was reduced.

### In-gel trypsin digestion

2-DE protein spots were excised from gels and treated as previously described [43]. They were trypsin digested using the Montage In-Gel DigestZP Kit (Millipore). The resulting peptides were analysed either by Matrix-Assisted Laser Desorption Ionisation - Time Of Flight (MALDI-TOF) MS or nano-liquid chromatography coupled to tandem mass spectrometry (nanoLC-MS/MS).

### Maldi-tof MS

A Biflex IV mass spectrometer (Bruker Daltonics) was used in reflectron mode for MALDI-TOF MS analysis of peptides and mass fingerprint identification of proteins. A saturated solution of α-cyano-4 hydroxycinnamic acid prepared in acetonitrile:water 1:1 with 0.1% trifluoroacetic acid was Â¼ diluted in the same solvent, and used as a matrix. Peptide and matrix solution were spotted on a stainless steel plate and air-dried. Calibration was done using a standard peptide mixture (Peptide Calibration Standard) from Bruker Daltonics as previously described [68]. Spectra were acquired over the 500–3500 *m/z* range by accumulating spectra from at least 100 shots, with ion extraction mode using an accelerating voltage of 19 kV and an extraction delay of 0.2 ns. Peptide mass fingerprints were analysed using the Mascot software version 2.2 (Matrix Science) and a local database comprising 3,455 *D. deserti* protein sequences totalling 980,690 amino acids [4]. The maximum number of miss-cleavages was set at one. Carbamidomethylation of cysteines was set as fixed modification and oxidation of methionines as variable modification. Protein identification reliability was evaluated on the basis of the probability-based Mowse score (p<0.05), mass error, number of peptides matches and similarity of experimental and theoretical protein molecular masses and isoelectric points.

### NanoLC-MS/MS

NanoLC-MS/MS was done either with an Esquire ion trap (Bruker Daltonics) coupled to an Ultimate™ NanoLC system (Dionex-LC Packings) or an LTQ-Orbitrap XL instrument (Thermo) coupled to an Ultimate 3000 LC-system (Dionex-LC Packings). In the former case, the instrument was controlled by HyStar TM software and on-line connected with a PepMap C18 nanocolumn (75 μm × 150 LC Packings). Samples (20 μL) were injected and separated with a 30 min linear gradient. The gradient was achieved from 5% solvent A (95:5 H_2_O/ACN 0.1%Fa) to solvent 50% B (20:80 H_2_O/ACN 0.1% FA) at a flow rate of 20 nl/min. Eluted peptides were electro-sprayed into the nano-ESI source of an Esquire 3000 Plus (Bruker). Spray voltage was set at 2000 V and capillary temperature at 170°C. The ion trap was operated in data-dependent MS to MS/MS switching mode using various precursors detected in the 100–2000 *m/z* unit window, scan resolution 13 000 *m/z* per second, selected using a 3 *m/z* unit ion isolation window and excluding single charged ions. Data were processed with the DataAnalysis software (Bruker Daltonics). Mgf files were searched against the *D. deserti* protein sequence local database using MASCOT 2.2 software (MatrixScience). MASCOT search parameters used with MS/MS data were similar as for those described above except: peptide tolerance at 0.4 Da, MS/MS tolerance at 0.4 Da, peptide charge at +1/+2/+3. Only peptides on rank 1 with an ion score above 30 were selected. Proteins identified by at least 2 different peptides were validated. Otherwise, LC-MS/MS experiments were performed on a LTQ-Orbitrap XL hybrid mass spectrometer (ThermoFisher) coupled to a UltiMate 3000 LC system (Dionex-LC Packings) in conditions similar as those previously described [69]. Briefly, peptide mixtures (0.1-2 pmol) were loaded and desalted online in a reverse phase precolumn Acclaim Pepmap 100 C18 (5μm bead size, 100 Å pore size, 5mm × 300 μm) from LC Packings. They were resolved on a nanoscale Acclaim Pepmap 100 C18 (3 μm bead size, 100Å pore size, 15 cm × 75 μm) from LC Packings at a flow rate of 0.3 μL/min. Peptides were separated using a 30 min-gradient (5 to 60% solvent B) with aqueous solvent A (0.1% HCOOH) and solvent B (0.1% HCOOH/80% CH_3_CN). The column was then washed for 10 min with 100% B and re-equilibrated for 20 min with 5% B. Full-scan mass spectra were measured from *m/z* 300 to 1800. The LTQ-orbitrap XL mass spectrometer was operated in the data-dependent mode using the TOP5 strategy with a Fourier Transform Mass Spectrometer (FTMS) resolution sets at 30,000 as previously reported [4]. In brief, a scan cycle was initiated with a full scan of high mass accuracy in the Orbitrap, which was followed by MS/MS scans in the linear ion trap on the 5 most abundant precursor ions with dynamic exclusion of previously selected ions. This dynamic exclusion consisted in two acquisitions of MS/MS spectra of the most abundant ion during a period of 30 sec and then excluding this ion for the followed fragmentations during the next 60 sec. The activation type was collisional-induced dissociation with a standard normalized collision energy sets at 30.

### Construction and radiation survival of *D. deserti* mutant strains

The *Deide_19260* deletion mutant was constructed using recently developed genetic tools for *D. deserti*[38]. Briefly, the mutant was obtained by allelic replacement via double homologous recombination after transformation of *D. deserti* wild-type with a plasmid construct derived from *E. coli* vector pUC19, which does not replicate in *D. deserti*. In this plasmid, one-kb fragments corresponding to DNA upstream and downstream of *Deide_19260* were cloned in the correct orientation respectively upstream and downstream of a kanamycin resistance cassette. The latter was cloned as a BamHI-PstI fragment in pUC19. Expression of the kanamycin resistance gene in this cassette is driven by the constitutive promoter of the *tuf* gene (*Deide_18970*, encoding elongation factor EF-Tu). After three rounds of selection on plates containing 10 μg/ml kanamycin, diagnostic PCR to confirm double homologous recombination at the correct locus and complete absence of *Deide_19260* was performed using appropriate primers. The *Deide_19260* deletion mutant was called SF7. The *Deide_20140* mutant, called SF8, was constructed in a similar way (but here the kanamycin cassette was present in pUC19 as a BamHI-XbaI fragment). All primer sequences are listed in Additional file 6: Table S3. Survival of mutants and wild-type after exposure to increasing doses of gamma (up to 10 kGy) or UV (up to 750 J/m^2^) irradiation was determined as described [32].

## Abbreviations

PFGE: Pulsed-Field Gel Electrophoresis; HCA: Hierarchical Clustering Analysis.

## Competing interests

The authors declare that they have no competing interests.

## Authors’ contributions

AD designed the experiments, analysed and interpreted the data, and drafted the manuscript. ES and PG recorded the proteomic and mass spectrometry data, and participated in the data interpretation. LB, SF and AdG prepared the microbial samples and performed the genetic experiments, BM performed the HCA analysis. AdG and JA conceived the study, participated in the data interpretation, drafted and revised the manuscript. All authors read and approved the final manuscript.

## Supplementary Material

Additional file 1 **Figure S1. **Reference 2D electrophoresis gel map for exponential *Deinococcus deserti* VCD115 cells. This map was established for cells grown at 30°C and harvested at the exponential phase. From 863 clearly distinguishable Coomassie-stained spots, we selected 131 spots among the most intense that could serve as landmarks. They are labelled on the figure and their characteristics reported in Additional file 2: Table S1. Click here for file

Additional file 2 **Table S1. **List of proteins of the reference 2DE-gel map This table lists the 171 soluble polypeptides unambiguously identified from 137 spots selected among the most intense. We can note the presence of 20 hypothetical conserved proteins and two of them highly expressed (Deide_06180 and Deide_11730). The most abundant proteins appear in bold face. Click here for file

Additional file 3 **Figure S2. **Post-irradiation growth of *D. deserti*. Click here for file

Additional file 4 **Figure S3. **Comparison between *Deide_1p01260* &*Deide_3p00210* genes, and RecA_C_ & RecA_P_ polypeptides. Panel A shows the sequence of Deide_1p01260 and Deide_3p00210 open reading frames with identical nucleotides in black and differing nucleotides in red. These different nucleotides are all in the third position of the codon and are silent mutations. Panel B shows the amino acid sequences of RecA_C_ (upper line) and RecA_P_ (bottom line). Amino acids that are different in these sequences are in blue and red respectively. Proteotypic tryptic peptides detected by mass spectrometry are indicated with blue and red lines. Click here for file

Additional file 5 **Table S2. **Comparison of radio-induced proteins of *D. deserti* with transcriptomics and proteomics data from *D. radiodurans.* Click here for file

Additional file 6 **Table S3. **Primers. Click here for file
