# Supplementary material for: Major soluble proteome changes in Deinococcus deserti over the earliest stages following gamma-ray irradiation
Source: Proteome Sci. 2013 Jan 15;11:3. doi: 10.1186/1477-5956-11-3 (PMC3564903; doi:10.1186/1477-5956-11-3)
Supplement: Additional file 1 — Figure S1. Reference 2D electrophoresis gel map for exponential Deinococcus deserti VCD115 cells. This map was established for cells grown at 30°C and harvested at the exponential phase. From 863 clearly distinguishable Coomassie-stained spots, we selected 131 spots among the most intense that could serve as landmarks. They are labelled on the figure and their characteristics reported in Additional file 2: Table S1. [file 1477-5956-11-3-S1.ppt]

## Slide 1
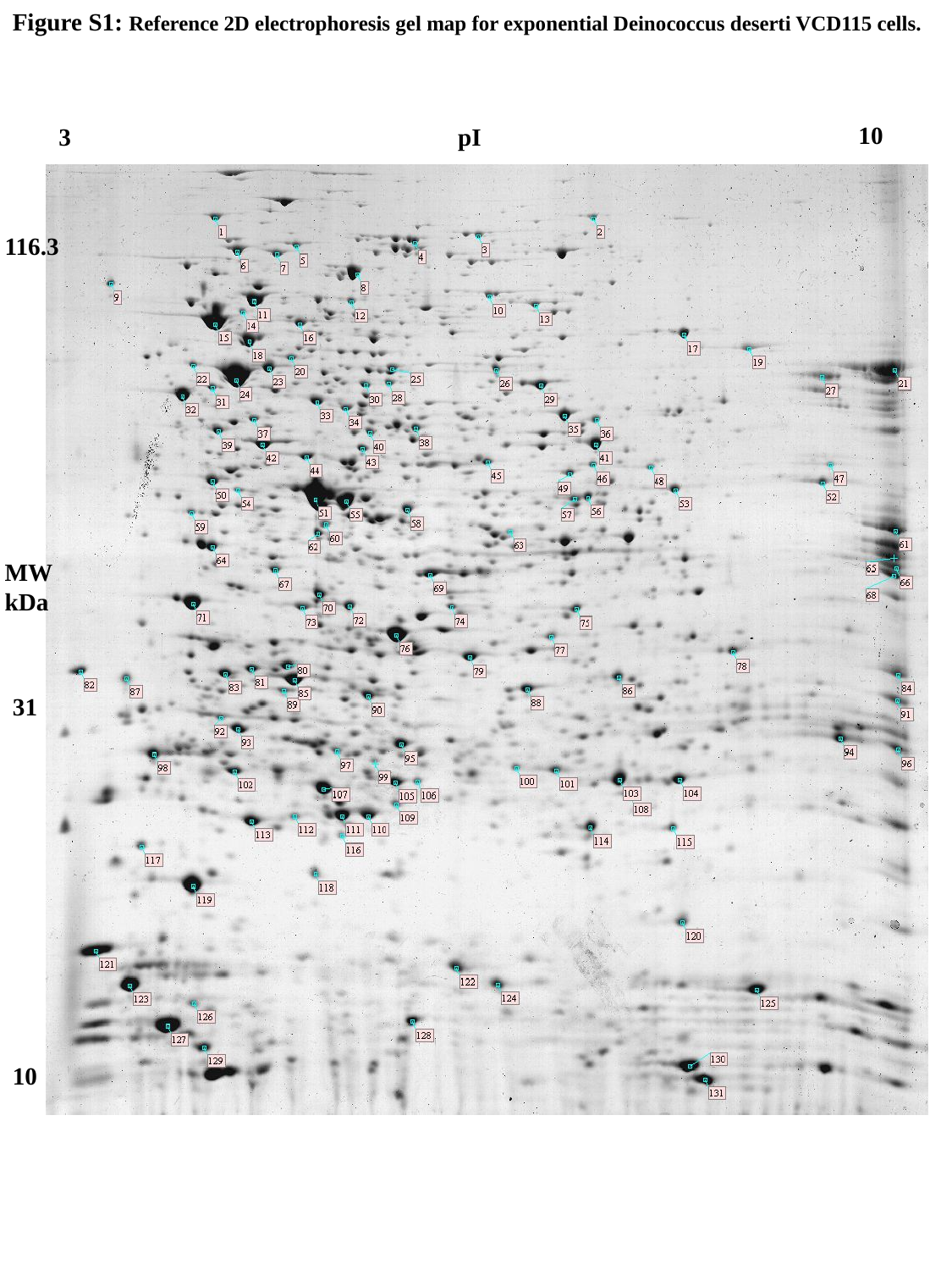

Figure S1: Reference 2D electrophoresis gel map for exponential Deinococcus deserti VCD115 cells.
10
3
pI
116.3
MW
kDa
31
10
