# Supplementary material for: Major soluble proteome changes in Deinococcus deserti over the earliest stages following gamma-ray irradiation
Source: Proteome Sci. 2013 Jan 15;11:3. doi: 10.1186/1477-5956-11-3 (PMC3564903; doi:10.1186/1477-5956-11-3)
Supplement: Additional file 2 — Table S1. List of proteins of the reference 2DE-gel map This table lists the 171 soluble polypeptides unambiguously identified from 137 spots selected among the most intense. We can note the presence of 20 hypothetical conserved proteins and two of them highly expressed (Deide_06180 and Deide_11730). The most abundant proteins appear in bold face. [file 1477-5956-11-3-S2.doc]

**Table S1** List of proteins of the reference map

| **Spot number** | **Acc. Number** | **Mascot score** | **MS Coverage** | **MW ex** | **pI exp.** | **MW calc.** | **pI calc.** | **Name** |
| --- | --- | --- | --- | --- | --- | --- | --- | --- |
|  |  |  |  |  |  |  |  |  |
| 1 | Deide_00700 | 961 | 29 | 98-188 | 4 - 4.8 | 112186 | 4.85 | Candidate Carbamoyl-phosphate synthase large chain |
| 2 | Deide_2p00780 | 359 | 13 | 98-188 | 5.8 - 6.6 | 118944 | 6.21 | Conserved hypothetical protein |
| 3 | Deide_06030 | 1145 | 32 | 98-188 | 5.4 - 5.6 | 127579 | 5.16 | Candidate DNA-directed RNA polymerase, beta subunit (RpoB) |
| 3 | Deide_21430 | 271 | 11 | 98-188 | 5.4 - 5.6 | 105294 | 5.75 | Candidate oxoglutarate dehydrogenase (succinyl-transferring) |
| 4 | Deide_04660 | 259 | 13 | 98-188 | 5,4 - 5,6 | 107715 | 5.57 | Related to ribonucleoside-diphosphate reductase |
| 4 | Deide_04940 | 314 | 12 | 98-188 | 5.4 - 5.6 | 103313 | 5.58 | Candidate glycine dehydrogenase [decarboxylating] (Glycine decarboxylase) |
| 5 | Deide_19270 | 403 | 14 | 62-98 | 4,8 - 5,2 | 96110 | 5.18 | Candidate alanine--tRNA ligase (Alanyl-tRNA synthetase) |
| 6 | Deide_07920 | 807 | 23 | 62-98 | 4.8 - 5.2 | 98284 | 4.95 | Candidate aconitate hydratase (citrate hydro-lyase) (aconitase) |
| 7 | Deide_17210 | 279 | 11 | 62-98 | 4,8 - 5,2 | 97160 | 5.1 | Candidate Preprotein translocase secA subunit |
| **8** | **Deide_08200** | **1259** | **44** | **62-98** | **5.2 - 5.4** | **91153** | **5.8** | **Related to Surface antigen precursor. outer membrane protein OMP85 family** |
| 9 | Deide_00370 | 246 | 2 | 62-98 | 3.0 - 4.0 | 46714 | 4.21 | Conserved hypothetical protein |
| 10 | Deide_12680 | 241 | 11 | 62-98 | 5.4 - 5.6 | 82283 | 5.9 | Candidate ATP-dependent Clp protease. ATP-binding subunit ClpC |
| 10 | Deide_18440 | 681 | 26 | 62-98 | 5.4 - 5.6 | 78042 | 5.83 | Candidate polyribonucleotide nucleotidyltransferase |
| **11** | **Deide_18990** | **1274** | **39** | **62-98** | **4.8 - 5.2** | **76643** | **5** | **Candidate elongation factor G, EF-G** |
| 12 | Deide_06410 | 593 | 27 | 62-98 | 5.2 - 5.4 | 73991 | 5.36 | Candidate threonine--tRNA ligase (threonyl-tRNA synthetase) |
| 13 | Deide_15490 | 444 | 23 | 62-98 | 5.6 - 5.8 | 74460 | 6.04 | Candidate DNA topoisomerase (ATP-hydrolyzing) (DNA gyrase, subunit B) |
| 14 | Deide_14930 | 101 | 9 | 62-98 | 4.8 - 5.2 | 77719 | 5.03 | Candidate Oligopeptidase A |
| **15** | **Deide_05790** | **1126** | **44** | **62-98** | **4 - 4.8** | **62699** | **4.89** | **Candidate ribosomal protein S1** |
| 16 | Deide_11340 | 235 | 16 | 62-98 | 4.8 - 5.2 | 66044 | 5.15 | Candidate GTP-binding TypA/BipA protein |
| 17 | Deide_09500 | 504 | 40 | 62-98 | 6.6 - 8 | 41500 | 9.62 | Candidate Maltose ABC transporter, periplasmic component |
| **18** | **Deide_00990** | **1377** | **44** | **62-98** | **4.8 - 5.2** | **63501** | **5.06** | **Candidate V-type ATP synthase alpha chain, (V-type ATPase subunit A)** |
| 19 | Deide_16800 | 841 | 37 | 62-98 | 6.6-8 | 65183 | 7.9 | Candidate DEAD-box ATP-dependent RNA helicase 3 |
| 20 | Deide_23470 | 238 | 9 | 49-62 | 4.8 - 5.2 | 53779 | 5.19 | Candidate Succinyl-CoA:dihydrolipoamide S-succinyltransferase |
| **21** | **Deide_12300** | **326** | **25** | **49-62** | **9.0 - 10** | **38609** | **9.49** | **Candidate Basic membrane protein family protein, precursor** |
| 22 | Deide_07350 | 343 | 21 | 49-62 | 4 - 4.8 | 63531 | 4.76 | Candidate maltose alpha-D-glucosyltransferase (trehalose synthase) |
| 23 | Deide_22230 | 759 | 26 | 49-62 | 4.8 - 5.2 | 73391 | 5.23 | Candidate subtilase-type serine protease precursor |
| 23 | Deide_1p00220 | 199 | 11 | 49-62 | 4.8 - 5.2 | 41589 | 4.9 | Candidate RNA polymerase sigma factor, sigma-70 family |
| **24** | **Deide_22590** | **2713** | **59** | **49-62** | **4.8 - 5.2** | **57827** | **4.96** | **Candidate 60 kDa chaperonin (Protein Cpn60) (groEL protein)** |
| 25 | Deide_16230 | 582 | 31 | 49-62 | 5.2 - 5.4 | 60039 | 5.47 | Candidate CTP synthase (UTP--ammonia ligase) (CTP synthetase) |
| 26 | Deide_12450 | 609 | 27 | 49-62 | 5.6 - 5.8 | 64498 | 5.95 | Candidate Succinate dehydrogenase, flavoprotein subunit |
| 27 | Deide_11101 | 404 | 32 | 49-62 | 8.0 - 9.0 | 46921 | 9.06 | conserved hypothetical protein, precursor |
| 28 | Deide_17500 | 425 | 28 | 49-62 | 5.2 - 5.4 | 56812 | 5.48 | Candidate glycine--tRNA ligase (Glycyl-tRNA synthetase) |
| 29 | Deide_09030 | 941 | 28 | 49-62 | 5.6 - 5.8 | 55933 | 5.87 | Candidate L-lysine 2,3-aminomutase |
| 30 | Deide_13670 | 431 | 27 | 49-62 | 5.2 - 5.4 | 58263 | 5.37 | Candidate 1-pyrroline-5-carboxylate dehydrogenase |
| 31 | Deide_03340 | 1059 | 34 | 49-62 | 4 - 4.8 | 52556 | 4.87 | Conserved hypothetical protein |
| 32 | Deide_05690 | 1196 | 50 | 49-62 | 4 - 4.8 | 49273 | 4.74 | Candidate Trigger factor, TF |
| 33 | Deide_21440 | 499 | 25 | 49-62 | 5.2 - 5.4 | 45567 | 5.34 | Candidate dihydrolipoyllysine-residue succinyltransferase |
| 34 | Deide_05580 | 620 | 36 | 49-62 | 5.2 - 5.4 | 56401 | 5.38 | Candidate GMP synthase |
| 35 | Deide_2p00580 | 632 | 31 | 49-62 | 5.6 - 5.8 | 55308 | 6.22 | Candidate 5-nucleotidase, precursor |
| 36 | Deide_19110 | 463 | 30 | 49-62 | 5.6 - 5.8 | 62903 | 9.14 | Related to peptide ABC transporter, periplasmic component |
| 37 | Deide_03280 | 226 | 24 | 49-62 | 4.8 - 5.2 | 49160 | 5.91 | Candidate Biotin carboxylase; (Acetyl-CoA carboxylase subunit A) |
| 38 | Deide_00040 | 828 | 28 | 49-62 | 5.4 - 5.6 | 51702 | 5.56 | Candidate Pyruvate kinase |
| 38 | Deide_21120 | 127 | 12 | 49-62 | 5.4 - 5.6 | 51953 | 5.62 | Related to 2-Nitropropane dioxygenase |
| 39 | Deide_10380 | 397 | 28 | 49-62 | 4.8 - 5.2 | 43808 | 4.93 | Candidate Transcription elongation factor NusA |
| 40 | Deide_2p00220 | 620 | 26 | 49-62 | 5.2 - 5.4 | 54959 | 5.43 | Candidate Glycerol kinase |
| **41** | **Deide_09040** | **998** | **36** | **49-62** | **5.6 - 5.8** | **49765** | **6.31** | **Candidate 4-aminobutyrate aminotransferase, aminotransferase class-III** |
| 42 | Deide_01000 | 645 | 33 | 49-62 | 4.8 - 5.2 | 51414 | 5.16 | Candidate V-type ATP synthase beta chain, V-type ATPase subunit B |
| 42 | Deide_08620 | 188 | 12 | 49-62 | 4.8 - 5.2 | 50174 | 5.15 | Candidate Glutamyl-tRNA (Gln) amidotransferase subunit A |
| 43 | Deide_19790 | 417 | 27 | 49-62 | 5.2 - 5.4 | 44019 | 5.35 | Candidate S-adenosylmethionine synthetase |
| 44 | Deide_03740 | 130 | 18 | 49-62 | 4.8 - 5.2 | 45578 | 5.2 | Candidate Histidinol dehydrogenase, (HDH) |
| 44 | Deide_18120 | 712 | 37 | 49-62 | 4.8 - 5.2 | 44779 | 5.23 | Candidate dihydroorotase (DHOase) |
| 45 | Deide_2p01640 | 385 | 33 | 38-49 | 5.4 - 5.6 | 39500 | 5.75 | Candidate cobW |
| 46 | Deide_11640 | 405 | 14 | 49-62 | 5.8 - 6.6 | 47102 | 6.36 | Candidate transcription termination factor rho |
| 47 | Deide_11100 | 316 | 26 | 28-38 | 5.4 - 5.6 | 38213 | 7.12 | Candidate ABC transporter, ATP-binding component |
| 47 | Deide_13180 | 501 | 42 | 28-38 | 5.4 - 5.6 | 28518 | 5.61 | Candidate 30S ribosomal protein S2 |
| 48 | Deide_03350 | 540 | 49 | 38-49 | 5.8 - 6.6 | 28252 | 4.88 | Candidate SUF system FeS assembly ATPase |
| 48 | Deide_05270 | 198 | 23 | 38-49 | 5.8 - 6.6 | 45078 | 6.7 | Related to toxic anion resistance protein |
| 48 | Deide_13360 | 162 | 16 | 38-49 | 5.8 - 6.6 | 27868 | 4.8 | Related to short-chain dehydrogenases |
| 49 | Deide_08810 | 659 | 40 | 38-49 | 5.6 - 5.8 | 52991 | 6.53 | Candidate Glycosyl hydrolase, family 13, precursor |
| 50 | Deide_18630 | 746 | 37 | 38-49 | 4 - 4.8 | 36306 | 4.87 | Candidate DNA-directed RNA polymerase alpha chain |
| 51 | Deide_18260 | 144 | 7 | 38-49 | 4.8 - 5.2 | 44603 | 5.13 | Candidate glutamate dehydrogenase |
| **51** | **Deide_06120 (=Deide_18970)** | **1132** | **45** | **38-49** | **4.8 - 5.2** | **44422** | **5.17** | **Candidate Elongation factor Tu, EF-Tu (TufA)** |
| **51** | **Deide_18970 (=Deide_06120)** | **1132** | **45** | **38-49** | **4.8 - 5.2** | **44422** | **5.17** | **Candidate Elongation factor Tu, EF-Tu (TufB)** |
| 52 | Deide_10700 | 328 | 26 | 38-49 | 8.0 - 9.0 | 45792 | 9.14 | Related to C-terminal processing peptidase |
| 53 | Deide_07670 | 615 | 23 | 38-49 | 5.8 - 6.6 | 43973 | 6.77 | Candidate ABC transporter, ATP-binding component |
| 54 | Deide_03760 | 223 | 18 | 38-49 | 4.8 - 5.2 | 42179 | 5.07 | Candidate Phosphopentomutase, (Phosphodeoxyribomutase) |
| 54 | Deide_2p01230 | 147 | 8 | 38-49 | 4.8 - 5.2 | 42716 | 4.96 | Conserved hypothetical protein |
| 55 | Deide_05750 | 69 | 11 | 38-49 | 5.2 - 5.4 | 43308 | 5.34 | Candidate Beta-ketoacyl-acyl-carrier-protein synthase I |
| 55 | Deide_10600 | 416 | 30 | 38-49 | 5.2 - 5.4 | 36245 | 5.65 | Candidate PpiC-type peptidyl-prolyl cis-trans isomerase precursor |
| 55 | Deide_11710 | 261 | 24 | 38-49 | 5.2 - 5.4 | 41530 | 5.33 | Candidate succinate--CoA ligase (succinyl-CoA synthetase) beta subunit |
| 55 | Deide_14250 | 133 | 13 | 38-49 | 5.2 - 5.4 | 39270 | 5.51 | Candidate DNA replication and repair protein (RecF) |
| 55 | Deide_15910 | 275 | 21 | 38-49 | 5.2 - 5.4 | 46051 | 5.35 | Candidate Glutamate-1-semialdehyde 2,1-aminomutase |
| 56 | Deide_20930 | 245 | 12 | 38-49 | 5.9 - 6 | 44052 | 6.28 | Candidate glycine hydroxymethyltransferase |
| 57 | Deide_00810 | 283 | 18 | 38-49 | 5.8 - 6.6 | 44001 | 6.01 | Candidate Carbamoyl-phosphate synthase small chain |
| 57 | Deide_11310 | 374 | 25 | 38-49 | 5.8 - 6.6 | 46534 | 6.05 | Candidate Glucose-1-phosphate adenylyltransferase |
| 58 | Deide_05640 | 388 | 36 | 38-49 | 5.4 - 5.6 | 40141 | 5.6 | Candidate 3-deoxy-7-phosphoheptulonate synthase |
| 58 | Deide_22070 | 141 | 11 | 38-49 | 5.4 - 5.6 | 44346 | 5.51 | Related to aminotransferases |
| 59 | Deide_18990 | 302 | 11 | 38-49 | 4 - 4.8 | 76643 | 5 | Candidate elongation factor G, EF-G |
| 60 | Deide_07360 | 130 | 20 | 38-49 | 4.8 - 5.2 | 35827 | 5.23 | Candidate Fructose-bisphosphatase (Fructose-1,6-bisphosphatase) |
| 60 | Deide_19100 | 121 | 10 | 38-49 | 4.8 - 5.2 | 36830 | 5.22 | Candidate D-alanine--D-alanine ligase |
| 61 | Deide_07720 | 548 | 26 | 38-49 | 9.0 - 10 | 33840 | 9.73 | Candidate TRAP transporter, periplasmic component |
| 61 | Deide_20600 | 342 | 29 | 38-49 | 9.0 - 10 | 33824 | 4.69. | Candidate inorganic diphosphatase (pyrophosphate phosphohydrolase) |
| 62 | Deide_02400 | 92 | 18 | 38-49 | 4.8 - 5.2 | 39873 | 5.33 | Candidate Histidinol-phosphate aminotransferase |
| 62 | Deide_16830 | 460 | 41 | 38-49 | 4.8 - 5.2 | 36852 | 5.24 | Candidate ketol-acid reductoisomerase |
| 63 | Deide_16670 | 231 | 18 | 38-49 | 5.6 - 5.8 | 40193 | 5.78 | Candidate acetyl-CoA C-acetyltransferase (Acetoacetyl-CoA thiolase) |
| 63 | Deide_19450 | 500 | 37 | 38-49 | 5.6 - 5.8 | 37806 | 5.82 | Candidate recombinase A (RecA) |
| 64 | Deide_14590 | 664 | 27 | 38-49 | 4.0 - 4.8 | 37764 | 4.85 | Candidate Cell division protein ftsZ |
| 65 | Deide_02140 | 147 | 18 | 38-49 | 9.0 - 10 | 31750 | 9.66 | Candidate iron ABC transporter, periplasmic component |
| 65 | Deide_05001 | 98 | 11 | 38-49 | 9.0 - 10 | 36434 | 9.63 | conserved hypothetical protein, precursor |
| 65 | Deide_2p01550 | 226 | 22 | 38-49 | 9.0 - 10 | 31621 | 9.52 | Distantly related to vitamin B12-binding protein btuF, precursor |
| 66 | Deide_06380 | 759 | 35 | 38-49 | 9.0 - 10 | 23609 | 9.18 | Candidate translation initiation factor IF-3 |
| 67 | Deide_10570 | 439 | 34 | 38-49 | 4.8 - 5.2 | 36853 | 5.13 | Candidate Rod shape-determining protein MreB |
| 68 | Deide_06070 | 494 | 20 | 38-49 | 9.0 - 10 | 24419 | 9.55 | Candidate 50S ribosomal protein L1 |
| 69 | Deide_11420 | 623 | 39 | 38-49 | 5.4 - 5.6 | 35678 | 5.6 | Candidate glyceraldehyde-3-phosphate dehydrogenase (GAPDH) |
| 70 | Deide_08180 | 255 | 22 | 38-49 | 5.2 - 5.4 | 32625 | 5.28 | Related to minicell-associated protein DivIVA |
| 70 | Deide_11700 | 844 | 45 | 38-49 | 5.2 - 5.4 | 30861 | 5.2 | Candidate succinate--CoA ligase (succinyl-CoA synthetase) alpha subunit |
| **71** | **Deide_03310** | **604** | **32** | **28-38** | **4 - 4.8** | **25440** | **4.73** | **Candidate 50S ribosomal protein L25, (General stress protein CTC)** |
| 72 | Deide_21050 | 353 | 40 | 28-38 | 5.2 - 5.4 | 35245 | 5.4 | Related to aldo/keto reductase family protein |
| 73 | Deide_08670 | 172 | 27 | 28-38 | 4.8 - 5.2 | 32524 | 5.27 | Candidate Methyltransferase |
| 73 | Deide_20240 | 370 | 35 | 28-38 | 4.8 - 5.2 | 35276 | 5.15 | malate dehydrogenase (malic dehydrogenase) |
| 74 | Deide_18500 | 245 | 27 | 28-38 | 5.4 - 5.6 | 34278 | 5.77 | Candidate GTP-binding protein era homolog |
| 75 | Deide_07620 | 287 | 37 | 28-38 | 5.6 - 5.8 | 35750 | 6.2 | Candidate ribose-phosphate diphosphokinase |
| **76** | **Deide_13180** | **1642** | **50** | **28-38** | **5.2 - 5.4** | **28518** | **5.61** | **Candidate 30S ribosomal protein S2** |
| 76 | Deide_2P00070 | 213 | 32 | 28-38 | 5.2 - 5.4 | 30153 | 5.44 | Related to NAD-dependent epimerase/dehydratase |
| 77 | Deide_01710 | 65 | 14 | 28-38 | 5.6 - 5.8 | 32675 | 6.15 | Candidate Acetyl-coenzyme A carboxylase carboxyl transferase subunit beta |
| 78 | Deide_01710 | 129 | 31 | 28-38 | 6.6 - 8.0 | 32675 | 6.15 | Candidate Acetyl-coenzyme A carboxylase carboxyl transferase subunit beta |
| 79 | Deide_22060 | 933 | 55 | 28-38 | 5.4 - 5.6 | 32895 | 5.63 | Candidate Fructose-1,6-bisphosphate triosephosphate-lyase, Aldolase |
| 80 | Deide_14540 | 1033 | 48 | 28-38 | 4.8 - 5.2 | 27584 | 5.25 | Conserved hypothetical protein |
| 81 | Deide_22590 | 111 | 6 | 28-38 | 4.8 - 5.2 | 57827 | 4.95 | Candidate 60 kDa chaperonin (Protein Cpn60) (groEL protein) |
| 81 | Deide_3p01250 | 221 | 24 | 28-38 | 4.8 - 5.2 | 33108 | 5.16 | Candidate thiosulfate sulfurtransferase (Rhodanese) |
| 82 | Deide_19240 | 309 | 26 | 28-38 | 3 - 4 | 25620 | 4.77 | Conserved hypothetical protein |
| 83 | Deide_19340 | 604 | 59 | 28-38 | 4.8 - 5.2 | 32272 | 4.95 | Candidate thiosulfate sulfurtransferase (Rhodanese) |
| 83 | Deide_23190 | 182 | 26 | 28-38 | 4.8 - 5.2 | 27002 | 4.92 | Candidate guanosine monophosphate kinase |
| 84 | Deide_00100 | 428 | 61 | 28-38 | 9.0 - 10 | 16025 | 9.23 | Candidate 50S ribosomal protein L9 |
| **85** | **Deide_13190** | **1605** | **66** | **28-38** | **4.8 - 5.2** | **28846** | **5.29** | **Candidate Elongation factor Ts (EF-Ts)** |
| 86 | Deide_12460 | 123 | 11 | 28-38 | 5.8 - 6.6 | 29242 | 6.59 | Candidate Succinate dehydrogenase, iron-sulfur protein |
| 87 | Deide_16690 | 166 | 33 | 28-38 | 4.0 - 4.8 | 17551 | 4.41 | Conserved hypothetical protein |
| 87 | Deide_20550 | 63 | 14 | 28-38 | 4.0 - 4.8 | 22255 | 4.33 | Conserved hypothetical protein |
| 88 | Deide_01030 | 89 | 15 | 28-38 | 5.6 - 5.8 | 31058 | 6.05 | Candidate Glucose kinase |
| 88 | Deide_20660 | 222 | 17 | 28-38 | 5.6 - 5.8 | 24512 | 6.13 | Related to Rieske 2Fe-2S protein |
| 88 | Deide_2p00580 | 72 | 9 | 28-38 | 5.6 - 5.8 | 55308 | 6.22 | Candidate 5-nucleotidase, precursor |
| 89 | Deide_22950 | 233 | 23 | 28-38 | 4.8 - 5.2 | 27108 | 4.93 | Candidate 5/3-ribonucleotide phosphohydrolase |
| 90 | Deide_01810 | 434 | 44 | 28-38 | 5.2 - 5.4 | 27718 | 5.39 | Conserved hypothetical protein |
| 91 | Deide_00130 | 772 | 49 | 28-38 | 9.0 - 10 | 11728 | 8.86 | Candidate 30S ribosomal protein S6 |
| 92 | Deide_19820 | 518 | 60 | 28-38 | 4.8 - 5.2 | 24556 | 4.85 | Candidate phosphate transport system protein phoU homolog |
| 93 | Deide_07540 | 416 | 20 | 28-38 | 4.8 - 5.2 | 32583 | 5.24 | Related to Desiccation-associated protein, precursor |
| 93 | Deide_14700 | 320 | 32 | 28-38 | 4.8 - 5.2 | 24735 | 4.88 | conserved hypothetical protein |
| 94 | Deide_07310 | 81 | 24 | 28-38 | 9.0 - 10 | 14818 | 11.16 | Candidate 30S ribosomal protein S9 |
| 94 | Deide_09860 | 700 | 47 | 28-38 | 9.0 - 10 | 18672 | 9.38 | Related to outer membrane chaperone Skp (OmpH) precursor |
| 95 | Deide_04570 | 116 | 15 | 28-38 | 5.4 - 5.6 | 21641 | 5.74 | Candidate phenylacetic acid degradation protein |
| 95 | Deide_19640 | 760 | 40 | 28-38 | 5.4 - 5.6 | 27853 | 5.53 | Candidate enoyl-[acyl-carrier-protein] reductase (NADH) |
| 96 | Deide_3p01280 | 596 | 56 | 28-38 | 9.0 - 10 | 16008 | 9.54 | Conserved hypothetical protein, precursor |
| 97 | Deide_09450 | 664 | 37 | 28-38 | 5.2 - 5.4 | 25310 | 5.55 | Related to Phage shock protein A |
| 98 | Deide_03710 | 104 | 48 | 28-38 | 4.0 - 4.8 | 19034 | 4.55 | Conserved hypothetical protein; candidate membrane protein |
| 99 | Deide_01400 | 246 | 31 | 28-38 | 5.2 - 5.4 | 23926 | 5.37 | Candidate Short-chain dehydrogenase/reductase SDR precursor |
| 100 | Deide_2p01570 | 206 | 35 | 28-38 | 5.6 - 5.8 | 25486 | 5.9 | Candidate precorrin-6B methylase 1 |
| 101 | Deide_04730 | 31 | 10 | 28-38 | 5.6 - 5.8 | 25043 | 6.3 | Distantly related to oxidoreductases |
| 101 | Deide_19210 | 948 | 30 | 28-38 | 5.6 - 5.8 | 25077 | 6.08 | Candidate response regulator, OmpR |
| 102 | Deide_06090 | 217 | 34 | 28-38 | 4.8 - 5.2 | 20825 | 4.9 | Candidate transcription antitermination protein |
| 102 | Deide_20700 | 569 | 52 | 28-38 | 4.8 - 5.2 | 24242 | 4.98 | Candidate response regulator, NarL |
| 103 | Deide_02360 | 471 | 28 | 28-38 | 5.8 - 6.6 | 23393 | 6.52 | Candidate Cyclase/dehydrase |
| 103 | Deide_09080 | 79 | 22 | 28-38 | 5.8 - 6.6 | 24497 | 6.59 | Candidate Oxidoreductase, short-chain dehydrogenase/reductase family |
| 104 | Deide_02360 | 174 | 32 | 28-38 | 5.8 - 6.6 | 23393 | 6.64 | Candidate Cyclase/dehydrase |
| 104 | Deide_09850 | 64 | 16 | 28-38 | 5.8 - 6.6 | 22742 | 8.04 | conserved hypothetical protein |
| 105 | Deide_06660 | 126 | 6 | 28-38 | 5.2 - 5.4 | 21934 | 5.59 | Candidate thymidine kinase |
| 105 | Deide_19550 | 795 | 51 | 28-38 | 5.2 - 5.4 | 25462 | 5.32 | Candidate response regulator, OmpR |
| 106 | Deide_09530 | 134 | 34 | 28-38 | 5.2 - 5.4 | 26018 | 5.59 | Candidate Inosine phosphorylase |
| **107** | **Deide_02590** | **555** | **31** | **28-38** | **5.2 - 5.4** | **20897** | **5.31** | **Candidate Peptidyl-prolyl cis-trans isomerase** |
| 108 | Deide_2p01560 | 306 | 32 | 28-38 | 5.8 - 6.6 | 22721 | 6.67 | Candidate cob(I)yrinic acid a,c-diamide adenosyltransferase |
| 109 | Deide_10791 | 283 | 36 | 17-28 | 5.2 - 5.4 | 22339 | 5.49 | Candidate FMN reductase |
| 109 | Deide_19570 | 283 | 35 | 17-28 | 5.2 - 5.4 | 22727 | 5.5 | Candidate Endopeptidase Clp |
| **110** | **Deide_00960** | **2084** | **58** | **17-28** | **5.2 - 5.4** | **20391** | **5.47** | **Candidate V-type ATP synthase subunit E** |
| 111 | Deide_00960 | 132 | 11 | 17-28 | 5.2 - 5.4 | 20391 | 5.47 | Candidate V-type ATP synthase subunit E |
| 111 | Deide_07760 | 93 | 14 | 17-28 | 5.2 - 5.4 | 22934 | 5.43 | Candidate superoxide dismutase |
| 111 | Deide_18100 | 112 | 18 | 17-28 | 5.2 - 5.4 | 20221 | 5.26 | Candidate bifunctional protein PyrR (regul./Uracil phosphoribosyltransferase) |
| 112 | Deide_07760 | 76 | 15 | 17-28 | 4.8 - 5.2 | 22934 | 5.43 | Candidate superoxide dismutase |
| 112 | Deide_06120 (=Deide_18970) | 181 | 15 | 17-28 | 4.8 - 5.2 | 44422 | 5.16 | Candidate Elongation factor Tu, EF-Tu (TufB) |
| 112 | Deide_18970 (=Deide_06120) | 181 | 15 | 17-28 | 4.8 - 5.2 | 44422 | 5.16 | Candidate Elongation factor Tu, EF-Tu (TufA) |
| **113** | **Deide_03300** | **1083** | **48** | **17-28** | **4.8 - 5.2** | **20546** | **5.04** | **Candidate Elongation factor P, EF-P** |
| 114 | Deide_13210 | 580 | 54 | 17-38 | 5.8 - 6.6 | 20359 | 6.19 | Candidate Ribosome recycling factor (Ribosome-releasing factor) (RRF) |
| 115 | Deide_18240 | 578 | 42 | 17-38 | 5.8 - 6.6 | 20760 | 6.66 | Hypothetical protein; candidate membrane protein |
| 116 | Deide_19850 | 97 | 10 | 17-38 | 5.2 - 5.4 | 20971 | 5.36 | Candidate orotate phosphoribosyltransferase |
| 117 | Deide_12690 | 77 | 20 | 17-38 | 4.0 - 4.8 | 22991 | 4.8 | Conserved hypothetical protein, precursor |
| 118 | Deide_07180 | 369 | 30 | 17-38 | 4.8 - 5.2 | 17267 | 5.31 | Candidate transcription elongation factor |
| **119** | **Deide_06180** | **1719** | **45** | **17-28** | **4.0 - 4.8** | **20122** | **5.42** | **Conserved hypothetical protein, precursor** |
| 120 | Deide_08510 | 753 | 29 | 17-28 | 5.8 - 6.6 | 20507 | 6.85 | Related to desiccation-associated late embryogenesis abundant protein |
| **121** | **Deide_06180** | **392** | **34** | **14-17** | **3.0 - 4.0** | **20122** | **5.42** | **Conserved hypothetical protein, precursor** |
| **122** | **Deide_07830** | **773** | **43** | **14-17** | **5.4 - 5.6** | **14950** | **5.63** | **Candidate nucleoside-diphosphate kinase (Nucleoside-2-P kinase)** |
| **123** | **Deide_11730** | **1334** | **44** | **14-17** | **4 - 4.8** | **17700** | **4.51** | **conserved hypothetical protein** |
| 124 | Deide_01490 | 88 | 15 | 14-17 | 5.6 - 5.8 | 17748 | 5.75 | Candidate Arginine repressor |
| 124 | Deide_16090 | 297 | 52 | 14-17 | 5.6 - 5.8 | 15999 | 5.73 | Candidate Osmotically-inducible protein C (OsmC-like protein) |
| **125** | **Deide_06060** | **652** | **35** | **14-17** | **6.6 - 8** | **17364** | **7.9** | **Candidate 50S ribosomal protein L10** |
| 126 | Deide_08720 part. | 193 | 24 | 14-17 | 4 - 4.8 | 36203 | 9.69 | Related to S-layer-like array protein |
| 126 | Deide_20130 part. | 233 | 14 | 14-17 | 4 - 4.8 | 38192 | 9.22 | Related to S-layer-like array protein, precursor |
| 127 | Deide_06020 part. | 124 | 3 | 14-17 | 4 - 4.8 | 171152 | 5.47 | Candidate DNA-directed RNA polymerase, subunit beta (RpoC) |
| **127** | **Deide_06050** | **990** | **32** | **14-17** | **4 - 4.8** | **12499** | **4.7** | **Candidate 50S ribosomal protein L7/L12** |
| **127** | **Deide_11730** | **259** | **34** | **14-17** | **4 - 4.8** | **17700** | **4.51** | **conserved hypothetical protein** |
| 128 | Deide_16110 | 917 | 32 | 14-17 | 5.4-5.6 | 15716 | 5.69 | conserved hypothetical protein |
| 129 | Deide_18600 | 168 | 52 | 14-17 | 4 - 4.8 | 13366 | 4.88 | Candidate Thioredoxin |
| 130 | Deide_09930 | 73 | 54 | 14-17 | 6.6 - 8 | 9902 | 6.73 | Candidate Cold-shock DNA-binding domain protein |
| 131 | Deide_2p00490 (=Deide_3p00840) | 160 | 40 | 14-17 | 6.6 - 8 | 9519 | 6.58 | Candidate Cold shock protein |
| 131 | Deide_3p00840 (=Deide_2p00490) | 160 | 40 | 14-17 | 6.6 - 8 | 9519 | 6.58 | Candidate Cold shock protein |

This table lists the 170 soluble polypeptides unambiguously identified from 131 spots selected among the most intenses. We can note the presence of 20 hypothetical conserved proteins and two of them highly expressed (Deide_06180 and Deide_11730). The most abundant proteins appear in bold face.
