# Supplementary material for: Major soluble proteome changes in Deinococcus deserti over the earliest stages following gamma-ray irradiation
Source: Proteome Sci. 2013 Jan 15;11:3. doi: 10.1186/1477-5956-11-3 (PMC3564903; doi:10.1186/1477-5956-11-3)
Supplement: Additional file 3 — Figure S2. Post-irradiation growth of D. deserti. [file 1477-5956-11-3-S3.ppt]

## Slide 1
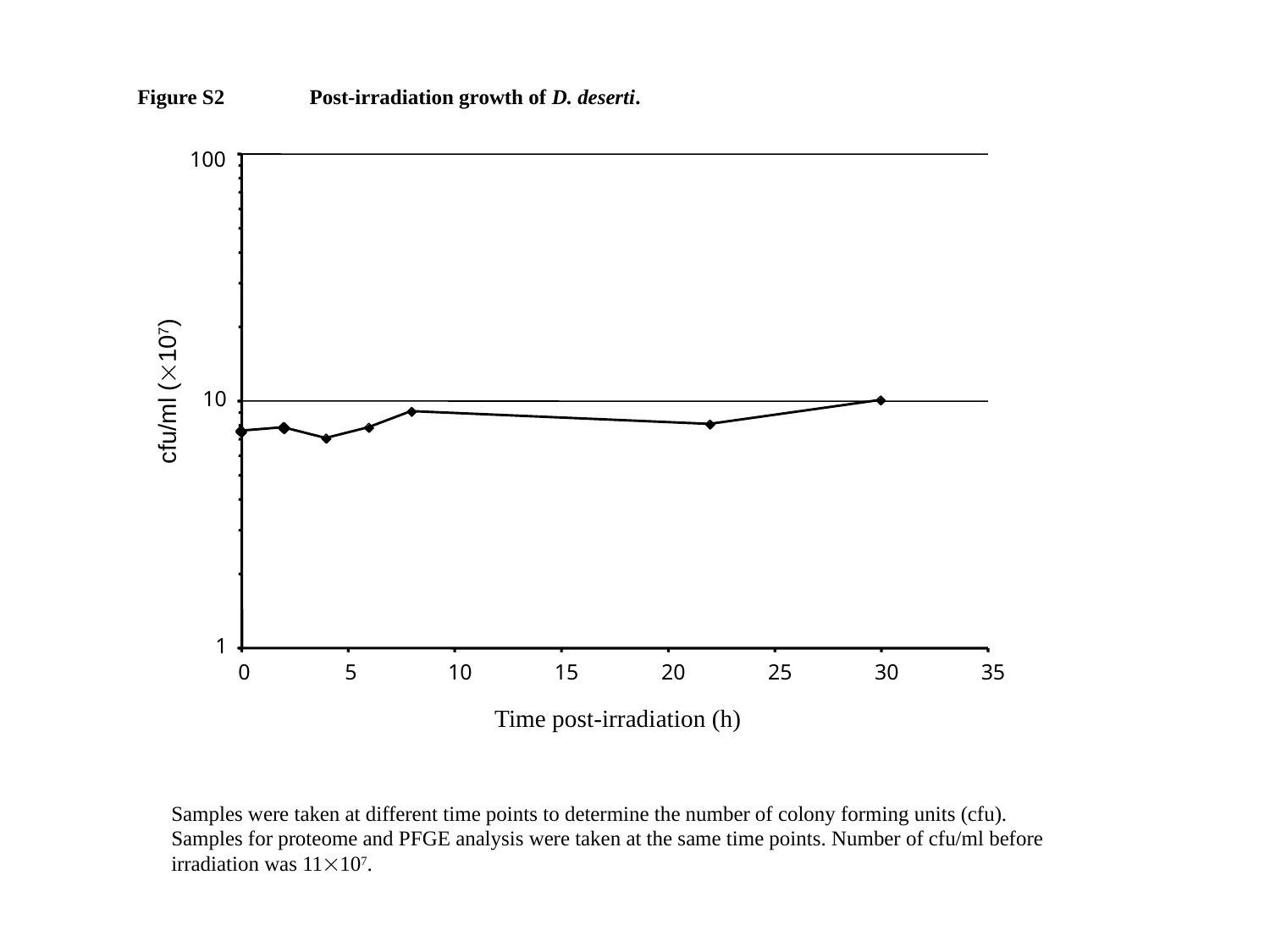

Figure S2 Post-irradiation growth of D. deserti.
100
cfu/ml (×107)
10
1
0
5
10
15
20
25
30
35
Time post-irradiation (h)
Samples were taken at different time points to determine the number of colony forming units (cfu). Samples for proteome and PFGE analysis were taken at the same time points. Number of cfu/ml before irradiation was 11×107.
