# Supplementary material for: Major soluble proteome changes in Deinococcus deserti over the earliest stages following gamma-ray irradiation
Source: Proteome Sci. 2013 Jan 15;11:3. doi: 10.1186/1477-5956-11-3 (PMC3564903; doi:10.1186/1477-5956-11-3)
Supplement: Additional file 4 — Figure S3. Comparison between Deide_1p01260 &Deide_3p00210 genes, and RecAC & RecAP polypeptides. Panel A shows the sequence of Deide_1p01260 and Deide_3p00210 open reading frames with identical nucleotides in black and differing nucleotides in red. These different nucleotides are all in the third position of the codon and are silent mutations. Panel B shows the amino acid sequences of RecAC (upper line) and RecAP (bottom line). Amino acids that are different in these sequences are in blue and red respectively. Proteotypic tryptic peptides detected by mass spectrometry are indicated with blue and red lines. [file 1477-5956-11-3-S4.ppt]

## Slide 1
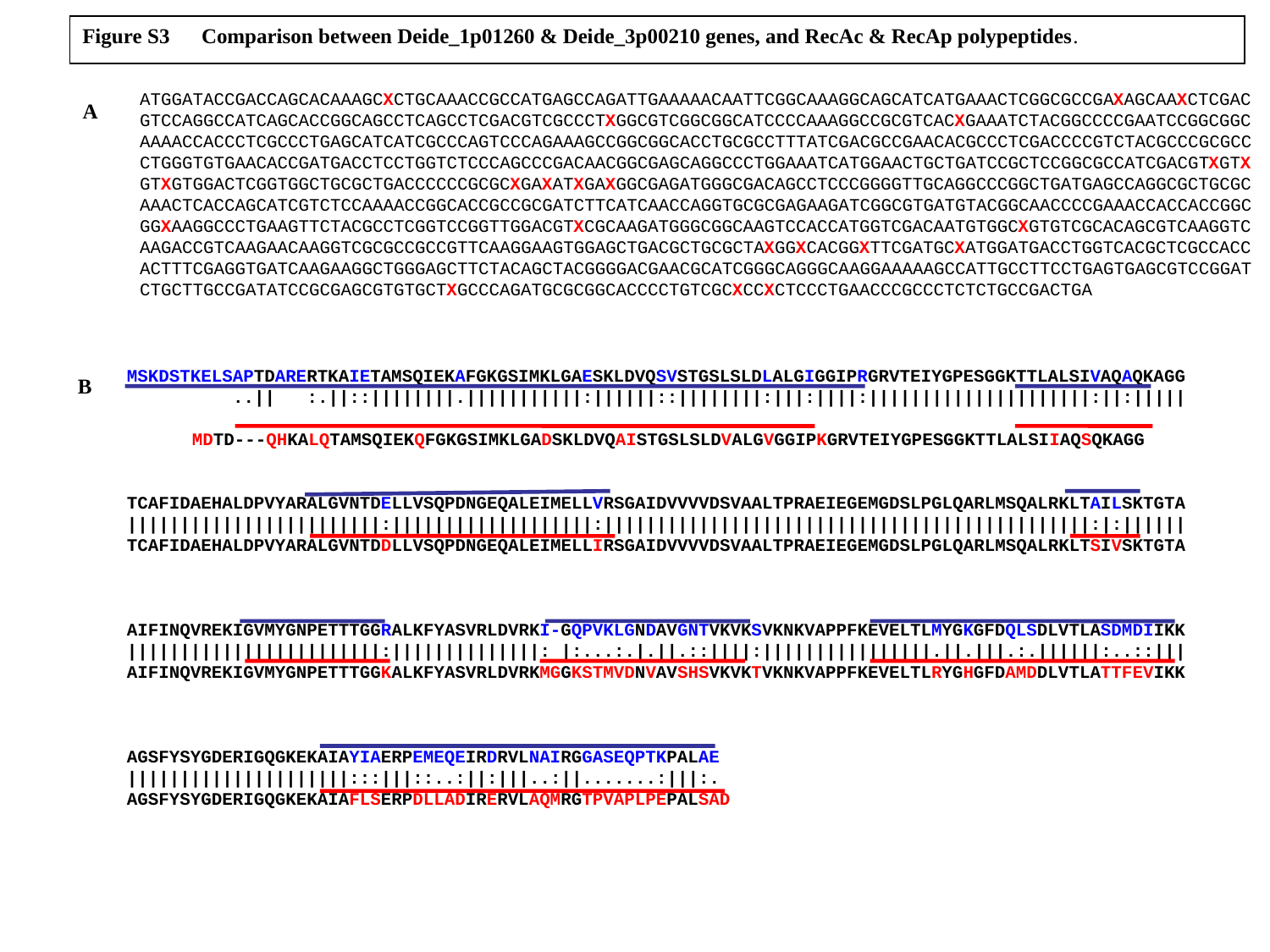

Figure S3 Comparison between Deide_1p01260 & Deide_3p00210 genes, and RecAc & RecAp polypeptides.
ATGGATACCGACCAGCACAAAGCXCTGCAAACCGCCATGAGCCAGATTGAAAAACAATTCGGCAAAGGCAGCATCATGAAACTCGGCGCCGAXAGCAAXCTCGACGTCCAGGCCATCAGCACCGGCAGCCTCAGCCTCGACGTCGCCCTXGGCGTCGGCGGCATCCCCAAAGGCCGCGTCACXGAAATCTACGGCCCCGAATCCGGCGGCAAAACCACCCTCGCCCTGAGCATCATCGCCCAGTCCCAGAAAGCCGGCGGCACCTGCGCCTTTATCGACGCCGAACACGCCCTCGACCCCGTCTACGCCCGCGCCCTGGGTGTGAACACCGATGACCTCCTGGTCTCCCAGCCCGACAACGGCGAGCAGGCCCTGGAAATCATGGAACTGCTGATCCGCTCCGGCGCCATCGACGTXGTXGTXGTGGACTCGGTGGCTGCGCTGACCCCCCGCGCXGAXATXGAXGGCGAGATGGGCGACAGCCTCCCGGGGTTGCAGGCCCGGCTGATGAGCCAGGCGCTGCGCAAACTCACCAGCATCGTCTCCAAAACCGGCACCGCCGCGATCTTCATCAACCAGGTGCGCGAGAAGATCGGCGTGATGTACGGCAACCCCGAAACCACCACCGGCGGXAAGGCCCTGAAGTTCTACGCCTCGGTCCGGTTGGACGTXCGCAAGATGGGCGGCAAGTCCACCATGGTCGACAATGTGGCXGTGTCGCACAGCGTCAAGGTCAAGACCGTCAAGAACAAGGTCGCGCCGCCGTTCAAGGAAGTGGAGCTGACGCTGCGCTAXGGXCACGGXTTCGATGCXATGGATGACCTGGTCACGCTCGCCACCACTTTCGAGGTGATCAAGAAGGCTGGGAGCTTCTACAGCTACGGGGACGAACGCATCGGGCAGGGCAAGGAAAAAGCCATTGCCTTCCTGAGTGAGCGTCCGGATCTGCTTGCCGATATCCGCGAGCGTGTGCTXGCCCAGATGCGCGGCACCCCTGTCGCXCCXCTCCCTGAACCCGCCCTCTCTGCCGACTGA
A
MSKDSTKELSAPTDARERTKAIETAMSQIEKAFGKGSIMKLGAESKLDVQSVSTGSLSLDLALGIGGIPRGRVTEIYGPESGGKTTLALSIVAQAQKAGG
 ..|| :.||::||||||||.|||||||||||:||||||::||||||||:|||:||||:|||||||||||||||||||||:||:|||||
 MDTD---QHKALQTAMSQIEKQFGKGSIMKLGADSKLDVQAISTGSLSLDVALGVGGIPKGRVTEIYGPESGGKTTLALSIIAQSQKAGG
TCAFIDAEHALDPVYARALGVNTDELLVSQPDNGEQALEIMELLVRSGAIDVVVVDSVAALTPRAEIEGEMGDSLPGLQARLMSQALRKLTAILSKTGTA
||||||||||||||||||||||||:|||||||||||||||||||:||||||||||||||||||||||||||||||||||||||||||||||:|:||||||
TCAFIDAEHALDPVYARALGVNTDDLLVSQPDNGEQALEIMELLIRSGAIDVVVVDSVAALTPRAEIEGEMGDSLPGLQARLMSQALRKLTSIVSKTGTA
AIFINQVREKIGVMYGNPETTTGGRALKFYASVRLDVRKI-GQPVKLGNDAVGNTVKVKSVKNKVAPPFKEVELTLMYGKGFDQLSDLVTLASDMDIIKK
||||||||||||||||||||||||:||||||||||||||: |:...:.|.||.::||||:||||||||||||||||.||.|||.:.||||||:..::|||
AIFINQVREKIGVMYGNPETTTGGKALKFYASVRLDVRKMGGKSTMVDNVAVSHSVKVKTVKNKVAPPFKEVELTLRYGHGFDAMDDLVTLATTFEVIKK
AGSFYSYGDERIGQGKEKAIAYIAERPEMEQEIRDRVLNAIRGGASEQPTKPALAE
|||||||||||||||||||||:::|||::..:||:|||..:||.......:|||:.
AGSFYSYGDERIGQGKEKAIAFLSERPDLLADIRERVLAQMRGTPVAPLPEPALSAD
B
