# Supplementary material for: Major soluble proteome changes in Deinococcus deserti over the earliest stages following gamma-ray irradiation
Source: Proteome Sci. 2013 Jan 15;11:3. doi: 10.1186/1477-5956-11-3 (PMC3564903; doi:10.1186/1477-5956-11-3)
Supplement: Additional file 5 — Table S2. Comparison of radio-induced proteins of D. deserti with transcriptomics and proteomics data from D. radiodurans. [file 1477-5956-11-3-S5.doc]

**Table S2. Comparison of radio-induced proteins of *D. deserti* with transcriptomics and proteomics data from *D. radiodurans***

| ***D. deserti* protein^a^** | ***D. radiodurans*** | | | | |
| --- | --- | --- | --- | --- | --- |
|  | transcriptomics | | proteomics | | |
|  | Tanaka 2004^b^ | Liu 2003^b^ | Basu 2012^b^ | Lu 2009^b^ | Zhang 2005^b^ |
| Deide_12520 (GyrA) (RDRM) (DR_1913) | + | + | + | - | - |
| Deide_15490 (GyrB) (RDRM) (DR_0906) | + | + | + | - | - |
| Deide_20140 (“MshD”) (DR_A0019) | - | - | - | - | - |
| Deide_19260 (RR, SarP) (DR_2556) | - | - | - | - | - |
| Deide_21840 (PilT) (DR_0442) | - | - | - | - | - |
| Deide_00120 (SSB) (RDRM) (DR_0099) | - | + | + | + | + |
| Deide_02990 (DdrB) (RDRM) (DR_0070) | + | + | + | - | - |
| Deide_2p01380 (PprA) (RDRM) (DR_A0346) | + | + | + | + | + |
| Deide_1p01260/Deide_3p00210 (RecA_P_) (RDRM) |  |  |  |  |  |
| Deide_19450 (RecA_C_) (RDRM) (DR_2340) | + | + | + | + | - |
| Deide_02842 (RDRM) (No homolog) |  |  |  |  |  |
| Deide_14090 (DR_1747) | - | + | - | - | - |
| Deide_13740 (FtsY) (DR_2260) | - | + | - | - | - |
| Deide_01160 (DdrD) (RDRM) (DR_0326) | + | Not tested | - | - | - |
| Deide_19590 (La, Lon) (DR_1974) | - | - | - | - | - |
| Deide_02310 (No homolog) |  |  |  |  |  |
| Deide_16440 (DR_0064) | - | - | - | - | - |
| Deide_12100 (UvrD) (RDRM) (DR_1775) | - | + | - | - | - |
| Deide_1p00780 (No homolog) |  |  |  |  |  |
| Deide_15960 (DR_1968) | - | - | - | - | - |
| Deide_23290 (EngA) (DR_2308) | - | + | - | - | - |

+, induced; -, not induced (or not detected as induced in proteome analyses)

^a^ Upregulated *D. deserti* proteins identified in this study. The *D. radiodurans* homolog is indicated in (red). The presence of the radiation/desiccation response motif upstream of a gene is indicated with (RDRM).

^b^ References: Tanaka et al, *Genetics* 2004, 168, 21-33; Liu et al, *Proc Natl Acad Sci U S A* 2003, 100, 4191-4196; Basu & Apte, *Mol Cell Proteomics* 2012, 11: M111.011734; Lu et al, *Mol Cell Proteomics* 2009, 8, 481-494; Zhang et al, *Proteomics* 2005, 5, 138-143.
