# Supplementary material for: Major soluble proteome changes in Deinococcus deserti over the earliest stages following gamma-ray irradiation
Source: Proteome Sci. 2013 Jan 15;11:3. doi: 10.1186/1477-5956-11-3 (PMC3564903; doi:10.1186/1477-5956-11-3)
Supplement: Additional file 6 — Table S3. Primers. [file 1477-5956-11-3-S6.doc]

**Table S3. Primers**

| **name** | **Sequence**^a^ | **use** |
| --- | --- | --- |
| **To amplify upstream and downstream DNA fragments for recombination** | | |
| 19260UpSac | GGAA**GAGCTC**GTGCTACCAGTTCAATGG | Upstream of Deide_19260 |
| 19260UpSma | GAGA**CCCGGG**ATTCCGGAGTCTAACAGC |
| 19260DnPstFw | TATA**CTGCAG**CGCGAGGCCTGGTGGGCT | Downstream of Deide_19260 |
| 19260DnPstRv | GAGA**CTGCAG**GCGCGTAGTTTGCCCTTC |
| 20140UpEco | GAGA**GAATTC**CTGAGGGGCTGGTGCAAG | Upstream of Deide_20140 |
| 20140UpSac | TATA**GAGCTC**CATCGGCACAATGGCGCA |
| 20140DnPst | TATA**CTGCAG**TGGGCCAGCTTCACCGAG | Downstream of Deide_20140 |
| 20140DnHd3 | GAGA**AAGCTT**GATCACGGACGGGGTGTG |
|  | | |
| **Diagnostic PCR** | | |
| Dd19260UpUp | AGGCTACGGGCCTGGTCGAG | Used with TufBamComp2 ^b^ |
| Dd19260DnDn | TCGCTATCTGAAGCGGCGAGA | Used with KanPstComp ^b^ |
| 20140UpUp | GACCAGGTCAAGGAAGGCGTTG | Used with TufBamComp2 ^b^ |
| 20140DnDn | CGGTGACCTGCGCGAGATCAG | Used with KanXbaCom ^b^ |
| TufBamComp2 | CTCAGCACGGCAATTACAAGGATCC |  |
| KanPstComp | CTTGACGAGTTCTTCTGACTGC |  |
| KanXbaCom | GCCTTCTTGACGAGTTCTTCTAATCTAGA |  |
| TufBam | GAA**GGATCC**TTGTAATTGCCGTGCTGAG | Kanamycin cassette (BamHI-PstI) |
| KanPst | GTT**CTGCAG**TCAGAAGAACTCGTCAAG |
| TufBam | GAA**GGATCC**TTGTAATTGCCGTGCTGAG | Kanamycin cassette (BamHI-XbaI) |
| KanXba | GG**TCTAGA**TTAGAAGAACTCGTCAAGAAGG |
| Dd19260FW | TGTTTCTGCCGGGTGCCGAC | Internal to Deide_19260 |
| Dd19260RV | CAATGGCCAGGGCACGCTGA |
| Dd20140FW | ACGCCCTTGAAGACGCTGCC | Internal to Deide_20140 |
| Dd20140RV | GACGGTCCAGGTGTGCAGGC |

^a^  Restriction sites in bold face

^b^ To confirm gene replacement by the kanamycin cassette at the correct locus
